# The evolution of place identity narratives: Long-term textual practices in the Chinese Baroque Historic Area

**DOI:** 10.1371/journal.pone.0359404

**Published:** 2026-10-05

**Authors:** Zongcheng Hu, Li Shao

**Affiliations:** School of Social Sciences, Harbin Institute of Technology, Harbin, China; Farhangian Teacher Education University: Farhangian University, IRAN, ISLAMIC REPUBLIC OF

## Abstract

In the processes of urban renewal and place branding, narratives of place identity undergo continuous evolution. As vital sites for local development, historic districts have seen their identity narratives become key strategies in urban marketing and spatial planning. However, existing research has overlooked the complexity of the dynamic evolution of place identity narratives, thereby limiting the effectiveness of planning, design, and communication in place branding. To systematically examine the long-term dynamic evolution of place identity narratives in historic districts, this study focuses on the Chinese Baroque Historic Area in Harbin, China. A total of 24 planning documents, news reports, and promotional texts from 2007 to 2024 were collected and categorized into three stages: the early stage, the middle stage, and the late stage. Subsequently, employing both quantitative analysis using the Latent Dirichlet Allocation (LDA) topic model and qualitative research methods, this study systematically reveals the characteristics of themes across different phases through dimensions such as information priority, structural dominance, and degree of dispersion. It further analyzes the evolutionary features of information structures across texts spanning these phases. The findings reveal a distinct phased evolution in the information structure of the place identity narrative for the Chinese Baroque Historic Area: the early stage was dominated by planning and policy documents, featuring highly concentrated information with institutional themes taking precedence. The middle stage saw a substantial increase in news and promotional texts, shifting the information structure from a high-concentration to a multi-thematic coexistence, accompanied by a redistribution of information priorities. The late stage is dominated by promotional and new media texts, in which themes of experience, image, and daily life gradually take a central position, leading to a relatively balanced information structure. Overall, the place identity narrative continuously evolves and gradually stabilizes through adjustments in information priority and restructuring of relational dynamics. The findings reveal the dynamic mechanisms and structural evolution of place identity narratives, providing important insights for advancing urban renewal and place branding practices.

## 1. Introduction

As competition among global cities intensifies, narratives of place identity have become increasingly crucial texts, repeatedly produced in urban planning and management, public governance, and social communication practices [1,2]. Against the backdrop of industrial restructuring and the transformation of urban development models, an increasing number of cities are leveraging place marketing and branding strategies to continuously enhance their image and regional appeal through diverse textual practices—including planning documents, news coverage, and promotional materials [3,4]. In this process, place identity is not merely a naturally formed cultural outcome but rather a narrative construct continually shaped, reconfigured, and stabilized through sustained textual production practices.

Although existing research has delved into the social significance of place identity narratives, multi-stakeholder participation mechanisms, and narrative strategies from perspectives such as human geography, urban studies, and place branding [5,6]. However, its analytical framework often implicitly assumes a linear generative hypothesis, tending to interpret place identity narratives as outcomes of rational design and natural operation. This perspective overlooks the inherent nature of place identity narratives as textual information systems. It also pays scant attention to their non-linear evolutionary characteristics and structural stabilization processes, which emerge through the continuous selection, reorganization, and compression of informational elements during the long-term, multi-stage production of texts. From the perspective of information science, the production of place identity narratives is simultaneously an outcome of information operations. The formation of place identity narratives does not originate from a single stage or a single type of text. Instead, it emerges through prolonged textual practices where information elements carried by texts from different stages undergo continuous adjustment and reorganization in terms of information priority, structural dominance, and degree of dispersion. Ultimately, certain information units attain relatively stable positions within the overall information structure. Therefore, it is necessary to place identity narratives within the analytical framework of long-term textual information systems, systematically examining their generation, evolution, and stabilization mechanisms from the perspective of information structure.

Moreover, as crucial sites for urban spatial reproduction and meaning encoding, the identity narratives of historic districts have become indispensable strategic tools in urban marketing and planning governance [7]. However, existing research generally treats place identity narratives as simplistic design outcomes, overlooking their non-linear evolutionary characteristics manifested through information selection, reorganization, and structural adjustments within long-term textual practices. This approach limits structural interpretations of place branding formation mechanisms and their communicative effects. Therefore, it is necessary to systematically examine the long-term dynamics of the generation of place identity narratives in historic districts from the perspective of information structure evolution. This approach will provide a more refined and structured theoretical framework for urban renewal and place branding practices.

Therefore, to systematically examine the long-term dynamic evolution of place identity narratives in historic districts, this study focuses on the Chinese Baroque Historic Area in Harbin, Heilongjiang Province, China. It conceptualizes the planning documents, news reports, and promotional materials generated during the district’s planning, construction, and promotion phases as a unified, continuously evolving textual information system. This study focuses on the organizational patterns of internal information structures within textual systems and their evolutionary processes across developmental stages. This study employs a quantitative analysis method based on the Latent Dirichlet Allocation (LDA) topic model, combined with qualitative interpretation, to identify latent information themes within texts from different stages [8,9]. This study further translates topic model outputs into information structure metrics to enable systematic analysis of the evolutionary characteristics of information structures within long-term text systems. The research findings reveal the long-term processes of formation and reconfiguration in the narratives of place identity in historic districts. This helps uncover their structural evolutionary patterns and provides more refined, systematic theoretical support for urban renewal and place branding practices.

Therefore, focusing on the information system formed by the long-term planning and marketing texts of the Chinese Baroque Historic Area, this study examines the mechanisms of generation, evolution, and stabilization of information structures across phased texts. It specifically explores the following three interrelated questions:

(1) What information structural characteristics does the text system related to the Chinese Baroque Historic Area exhibit at the overall level?
(2) What distinct informational functions do narrative texts from different phases perform in the construction of place identity narratives?
(3) How do thematic information elements undergo non-linear restructuring across phases through shifts in information priority and structural dominance?

## 2. Literature review

In the context of urban renewal and place branding, place identity research originated in human geography and environmental psychology, primarily focusing on how place identity is formed through spatial experiences and social memory [10,11]. With the rise of place marketing and city branding practices, place identity has gradually been incorporated into urban governance and development research, serving as a crucial narrative foundation for attracting tourists, capital, and external resources [12]. Within this research framework, place identity is no longer understood as a naturally occurring cultural outcome but rather as a crucial policy tool continuously shaped through planning and construction, narrative construction, and communication practices [13,14]. Throughout this process, narrative and storytelling approaches are widely regarded as crucial strategic tools for integrating local significance and addressing the specific cultural needs of consumer groups [6]. Accordingly, research on place identity narratives has moved beyond examining what cultural meanings are embedded in places and has increasingly focused on how complex, diverse, and sometimes conflicting elements of local information are selected, organized, and transformed into relatively coherent public representations. Existing studies have primarily examined the strategic construction and dissemination mechanisms of place identity narratives, as well as the exclusionary effects and sociopolitical consequences arising from selective representation, thereby advancing our understanding of the logic of narrative construction and its broader social implications. However, less attention has been paid to how diverse and competing elements of local information are continuously selected, reinforced, and reconfigured through long-term practices, eventually producing relatively stable structures of place identity narratives. Therefore, a systematic review of theoretical developments concerning the construction and critique of place identity narratives provides an essential foundation for further investigating their dynamic formation and long-term evolution.

Regarding the strategic construction and dissemination of place identity narratives, numerous studies indicate that, due to the inherent ambiguity and complexity of places, narratives of place identity often require selective expression and symbolic processing to simplify complex local elements and historical cultures into recognizable, communicable narratives [15]. In practice, urban administrators and marketing agencies typically select limited geographic features, cultural symbols, or historical fragments to represent the locality as a whole. By excluding discordant or “noisy” elements, they construct spatial imagery characterized by consistency and consumer appeal [16,17]. At the same time, the complex history and diverse meanings of local places are often compressed into specific time segments, forming selective memories and phased narrative structures [18]. Building on these insights, research on place storytelling has further developed a range of principles, frameworks, and communication models to enhance the practical applicability of narrative construction and improve its effectiveness in communication [19,20]. In addition, existing studies have examined how flagship architecture, iconic spaces, major events, and local cultural festivals contribute to strengthening the symbolic distinctiveness of places and enhancing their visibility among external audiences [21,22]. Existing studies have provided substantial insights into the design, packaging, and dissemination of place identity narratives. However, they have largely treated place identity narratives as outcomes characterized by relatively stable meanings and structures, while paying insufficient attention to the processes through which these narratives are formed. In particular, limited attention has been given to how different cultural information elements evolve, are adjusted, and are reconfigured across different stages of place branding practices. Therefore, further research is needed to examine the dynamic formation of place identity narratives and to reveal how diverse cultural information elements are continuously selected, reinforced, marginalized, and reorganized throughout place branding processes.

Cultural selection and the exclusion of particular meanings in place identity narratives are often closely intertwined with specific power relations and their broader sociopolitical consequences. Place-image construction led by governments, development agencies, or local elites tends to privilege positive elements that support economic growth, tourism development, and urban competitiveness, while downplaying or obscuring poverty, social deprivation, spatial inequality, and contested local histories [16,17]. For example, some urban nostalgia narratives selectively connect historical commercial traditions with visions of global urban development, reinforcing historical memories that are conducive to place branding and external promotion, while excluding or marginalizing controversial historical episodes, political movements, and the social costs associated with urban renewal projects [23]. In response to such top-down control over narrative production, participatory and collaborative approaches to place branding have shifted attention toward the co-construction of place meanings among diverse actors. They emphasize that stakeholders may hold different, and sometimes competing, interpretations of place meanings, and argue that continuous interaction, dialogue, and negotiation are essential for enhancing the democratic legitimacy and social inclusiveness of place identity narratives [24,25]. Therefore, place identity narratives should not be understood as value-neutral cultural representations; rather, they are selective processes embedded in struggles over meaning, resource allocation, and power relations. Clarifying the selection of information within place identity narratives and the underlying power dynamics provides a theoretical foundation for examining how information elements are added, removed, weighted, and structurally reorganized across different stages of place branding.

Overall, existing studies have examined the strategic construction of place identity narratives and their sociopolitical consequences from social, cultural, and governance perspectives, thereby advancing understanding of the mechanisms underlying narrative formation and its broader social implications. However, place identity narratives are not directly produced by a single event, text, or developmental stage; rather, they emerge as stage-specific structures formed through the continuous selection, translation of meanings, reinforcement, attenuation, and recombination of diverse local information elements throughout long-term processes of textual production. Traditional case studies, discourse analysis, and manual coding approaches have effectively explained cultural selection processes and the power relations underlying them. Nevertheless, these approaches are limited in their ability to systematically compare the coexistence and changing relative importance of multiple information elements across different stages of textual development, as well as to reveal how place identity narratives gradually acquire relatively stable structures through continuous processes of information adjustment. Therefore, understanding the dynamic formation of place identity narratives requires further attention to how information elements are organized, how structural relationships among them evolve, and how these mechanisms operate throughout long-term processes of textual production.

From an information science perspective, place identity narratives can be understood as organizational outcomes emerging from long-term textual information systems. They are not directly generated by a single text or developmental stage; rather, they gradually take shape through the continuous selection, translation, and reconfiguration of information during the production of multiple textual sources, including planning documents, news reports, and promotional materials. Different information elements exhibit varying levels of prominence across different stages, eventually contributing to the formation of relatively stable information structures. The Latent Dirichlet Allocation (LDA) model conceptualizes texts as probabilistic combinations of multiple latent topics, which aligns with the theoretical understanding that place identity narratives are composed of diverse information elements whose relative thematic weights continuously change over time. Compared with traditional content analysis approaches, LDA is capable of identifying latent thematic structures and translating processes of cultural selection and narrative restructuring into comparable distributions of topic probabilities. Although LDA has been widely applied in cultural tourism text analysis [26,27], existing studies have primarily focused on static topic identification, with limited attention paid to the processes of topic reinforcement, attenuation, and reconfiguration throughout long-term place branding practices. Furthermore, as important sites of local development, historic districts have increasingly incorporated identity narratives into urban marketing and spatial planning strategies. However, existing studies have paid insufficient attention to the complexity of the dynamic evolution of place identity narratives, thereby limiting the effectiveness of place branding planning, design, and communication. Therefore, this study employs LDA as a topic identification tool and integrates analyses of information priority and structural dominance to develop an analytical framework for long-term textual information structures, aiming to reveal the dynamic evolution of place identity narratives in historic districts.

Therefore, this study adopts an information structure perspective to analyze the evolutionary process of place identity narratives within a long-term, multi-stage textual system. By introducing the Latent Dirichlet Allocation (LDA) topic model, this study identifies latent information themes within texts related to the Chinese Baroque Historic Area. The outputs of the topic model are further translated into indicators of information structure, such as information priority, structural dominance, and degree of dispersion. This enables a systematic investigation of the non-linear restructuring process of information structures across different textual phases. This study aims to reveal how place identity narratives progressively achieve structural compression and stabilization through long-term textual practices, providing empirical support for research on the long-term evolution of complex social texts from an information science perspective.

## 3. Data and methods

### 3.1 Research area

Existing research on narratives of place identity predominantly focuses on large-scale spatial units such as cities or regions, relatively neglecting the long-term construction process of identity narratives within small-scale urban spaces [28,29]. At the same time, existing research predominantly adopts a cross-sectional analytical approach, focusing on short-term or individual project operations, and thus struggles to systematically reveal the evolutionary mechanisms through which place identity narratives undergo continuous adjustment, reorganization, and gradual stabilization across different stages [30,31]. In contrast, the identity narrative of the Chinese Baroque Historic Area did not emerge as a one-time outcome. Instead, it evolved through a prolonged, multi-stage process of sustained planning and governance practices. This trajectory offers a relatively comprehensive illustration of how place identity narratives form, adjust, and stabilize over extended periods of textual production. Therefore, this study selects the Chinese Baroque Historic Area as its research subject to explore the evolutionary mechanisms of information structures over the long term in texts.

The Chinese Baroque Historic Area is located in Harbin City, Heilongjiang Province, China. This district forms the core of Harbin’s old town and has witnessed the city’s developmental transformations. Following the construction of the China-Eastern Railway in 1896, Russia established a modern city with Western characteristics in Harbin. Thanks to the infusion of European civilization, Harbin rapidly transformed from a fishing village into a major metropolis in the Far East [32]. In the 1920s, Harbin emerged as a major international metropolis in Northeast Asia. The Daowai District, home to the Chinese Baroque Historic Area, experienced unprecedented socioeconomic prosperity, boasting hundreds of industries—including money exchanges, banks, grain trading, general merchandise, and export trade—and thousands of businesses. Many affluent Chinese businesspeople who have achieved commercial success have built buildings with Western-style facades to meet their operational needs while preserving the traditional Chinese courtyard layout in their floor plans. Furthermore, decorative details incorporate auspicious traditional symbols such as bats, pomegranates, endless knots, golden toads, and peonies [33]. Because these Sino-Western hybrid buildings share certain similarities with European Baroque architecture, a Japanese scholar coined the term “Chinese Baroque” for this architectural style in 1985.

After the 1990s, local commerce and trade gradually declined. Numerous old buildings deteriorated due to long-term neglect, while infrastructure and sanitation conditions progressively worsened. Ultimately, the neighborhood gradually devolved into an urban slum and an economically underdeveloped area. To advance tourism-driven commercial development and rebrand the city, the local government launched continuous preservation and renewal projects since 2000, renaming the revitalized district the “the Chinese Baroque Historic Area”. Through sustained planning, construction, and promotional efforts, the district has gradually transformed into a multifunctional space integrating historical and cultural displays, tourism consumption, and urban image-building. The renewed Chinese Baroque Historic Area is enclosed by Jingyu Street, Jingyang Street, Nanxun Street, and Nansidao Street, as shown in Fig 1. Based on its spatial organization and functional positioning, the district is divided into four thematic zones: a leisure and commercial zone between Jingyang Street and Nantoudao Street, a food and dining zone between Nantoudao Street and Nanerdao Street, a cultural and creative zone between Nanerdao Street and Nansandao Street, and a commercial residential and leisure zone between Nansandao Street and Nansidao Street. These functional zones, together with the major streets, historic buildings, cultural facilities, experiential nodes, and commercial clusters, constitute the post-renewal physical spatial framework of the district and support its transformation from a traditional residential area into a cultural tourism and consumption space. Currently, the Chinese Baroque Historic Area has become an important historical and cultural tourism destination in Harbin and has been designated as a National 3A-level Tourist Attraction.

**Fig 1:**
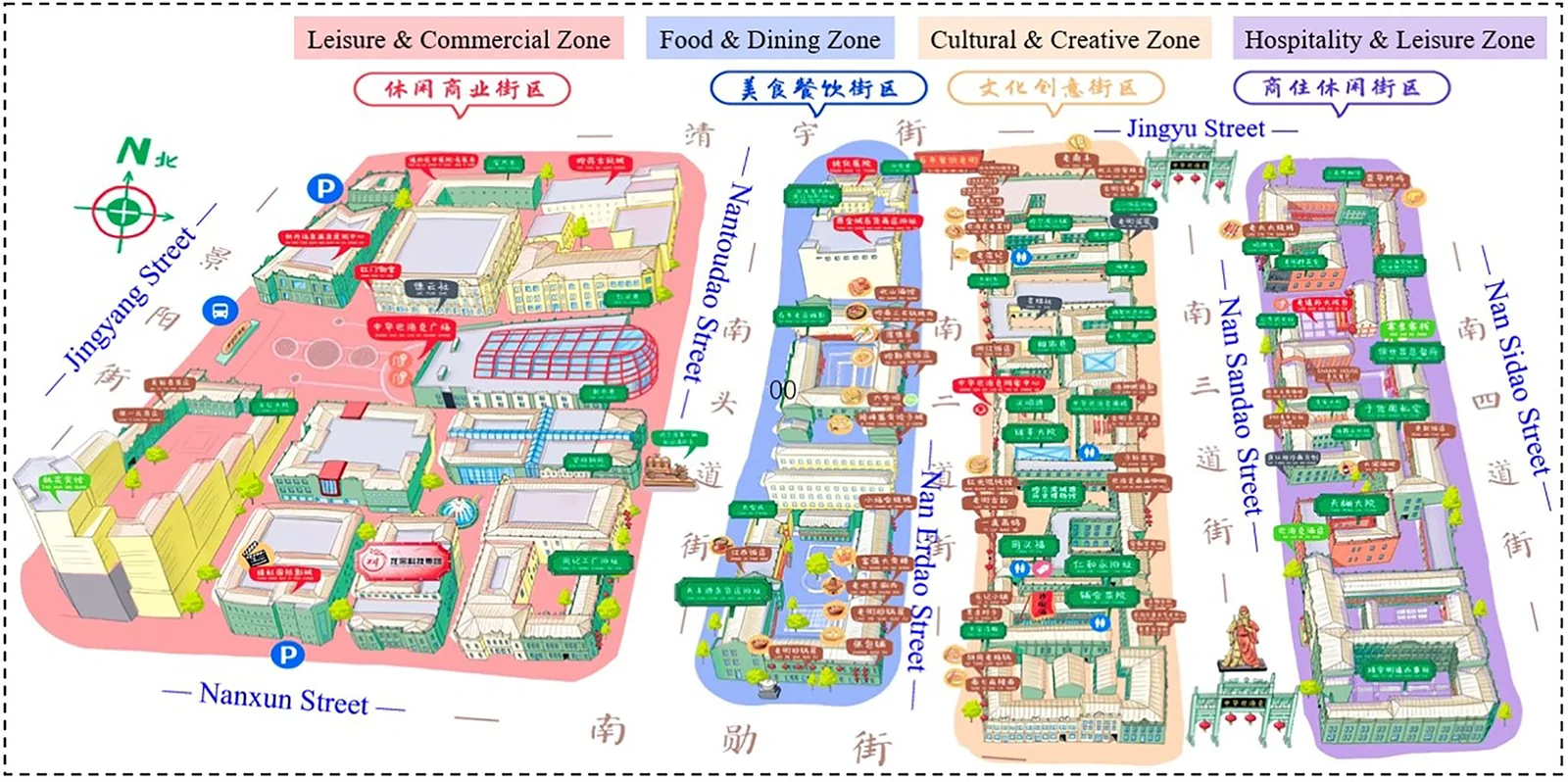
Spatial distribution and functional zoning of the Chinese Baroque Historic Area. Adapted from the original spatial guide map of the Chinese Baroque Historic Area, with modifications by the authors. The figure is similar but not identical to the original image and is presented for illustrative purposes only. Adapted under a CC BY 4.0 license with permission from Luyao Wang, original copyright 2023.

### 3.2 Research design and topic modeling

Place branding texts often exhibit high-dimensional lexical features and complex modes of expression, incorporating both institutionalized language from planning and policy documents and narrative expressions from news media and branding communications. This complexity makes traditional classification and clustering approaches based on lexical similarity insufficient for fully revealing the underlying semantic structures embedded within the texts. Topic models reconstruct the semantic space of the text through probabilistic generation mechanisms, providing a new analytical framework for identifying latent topic structures and semantic organization patterns [34,35]. Among these approaches, the Latent Dirichlet Allocation (LDA) model, as a probabilistic topic model, does not require predefined classification frameworks. Instead, it represents documents as probabilistic combinations of multiple topics with varying weights and automatically identifies latent thematic structures within a corpus, thereby improving the objectivity and reproducibility of comparative analyses across texts [36]. By reducing subjective bias arising from predefined thematic categories and establishing a link between computational analysis and qualitative interpretation, LDA has been widely applied in studies of policy documents, strategic texts, and cultural tourism texts, including research on destination image construction, the evolution of tourism discourse, and the analysis of cultural representations [37]. However, existing studies have primarily focused on static topic identification, paying limited attention to the dynamic processes through which themes are reinforced, weakened, and reconfigured throughout long-term place identity narrative practices. Meanwhile, conventional LDA typically treats documents as independent analytical units, which limits its ability to capture the structural characteristics of longitudinal corpora involving variations across temporal stages and text types. Therefore, this study

employs LDA as a topic identification tool and optimizes the number of topics using perplexity and topic coherence measures to improve the stability and interpretability of topic identification results. Based on the document–topic probability distributions generated by the LDA model, this study further extracts topic weights from texts across different stages, which serve as the foundation for subsequent information structure analysis. The overall analytical framework is presented in Fig 2.

**Fig 2:**
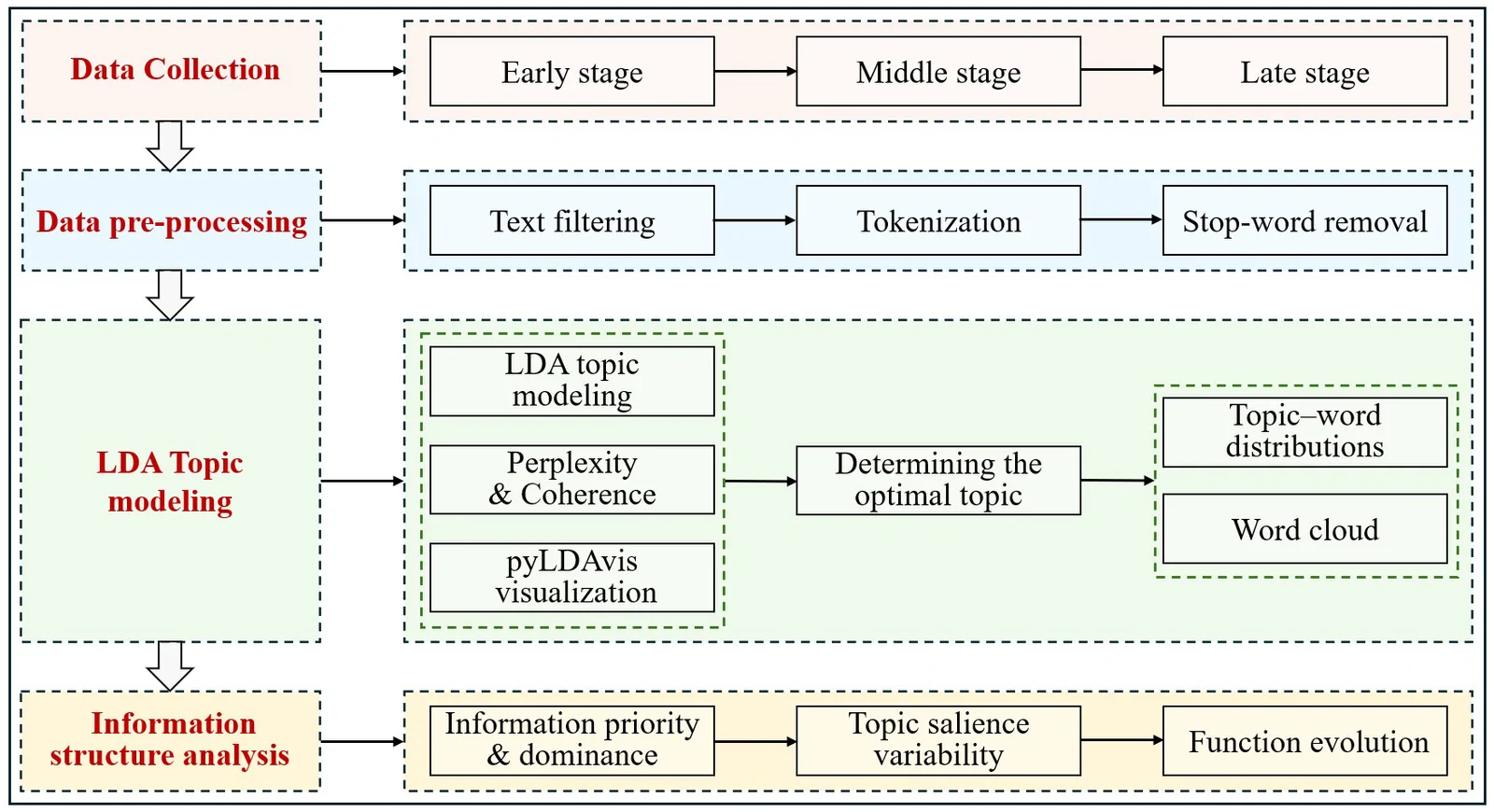
The overall analysis framework.

Texts related to place branding often exhibit high-dimensional lexical features and complex linguistic expressions, incorporating both the institutionalized discourse of planning and policy documents and the narrative-oriented expressions found in news communication and branding materials. This complexity makes traditional classification and clustering approaches based on lexical similarity insufficient for fully capturing the latent semantic structures embedded within these texts [34,35]. Topic models reconstruct the semantic space of texts through probabilistic generative mechanisms, providing a new analytical framework for identifying latent thematic structures and patterns of semantic organization. Among these methods, the Latent Dirichlet Allocation (LDA) model, as a probabilistic topic model, does not require predefined classification frameworks. Instead, it represents documents as combinations of multiple topics with different weights and automatically identifies latent thematic structures within a text corpus, thereby improving the objectivity and reproducibility of cross-text comparative analysis [38,39]. By reducing the subjectivity associated with predefined thematic categories and bridging computational analysis with qualitative interpretation, LDA has been widely applied in studies of policy documents, strategic texts, and cultural tourism materials, including research on destination image construction, the evolution of tourism discourse, and cultural representation analysis [40,41]. Moreover, previous studies of policy texts have extended topic-modeling results by incorporating quantitative measures of information priority, structural dominance, dispersion, and concentration to characterize the organization and distribution of thematic information within textual structures [42]. However, existing studies have primarily focused on static topic identification, with insufficient attention paid to the dynamic processes through which themes are reinforced, weakened, and reconfigured throughout long-term place identity narrative practices. Meanwhile, conventional LDA typically treats documents as independent analytical units, limiting its ability to explain the structural characteristics of longitudinal corpora involving variations across temporal stages and text types. Therefore, this study employs LDA as a topic identification tool and determines the optimal number of topics based on perplexity and topic coherence measures. Furthermore, it integrates information priority analysis, structural dominance evaluation, and keyword co-occurrence network analysis to establish a framework for analyzing long-term textual information structures, thereby revealing the dynamic evolution of place identity narratives during the place branding process of the Chinese Baroque Historic Area.

To reveal how key actors, including government departments, planning and construction institutions, and operational entities, select, interpret, and disseminate local cultural meanings through textual practices during the place branding process of historic districts, this study collects relevant texts generated throughout the renewal and branding process of the Chinese Baroque Historic Area in Harbin from 2007 to 2024. The textual corpus includes planning documents, policy-related materials, news reports, operational management documents, and marketing and promotional materials. It should be noted that this study does not aim to construct an annual textual time series or capture year-by-year changes in narratives. Instead, it focuses on the long-term evolution of information priorities, structural relationships, and modes of expression within place identity narratives across different development stages. Therefore, purposive sampling was employed to select texts based on their relevance to the district’s renewal and branding process, source reliability, and explanatory value for understanding the construction of place identity narratives. Specifically, texts were screened according to three criteria: relevance, non-duplication, and narrative completeness. Materials that were unrelated to the research topic, repeatedly reproduced, or lacking substantive narrative content were excluded, while texts reflecting processes of place identity narrative construction, including historical-cultural representation, spatial image formation, functional transformation, and brand communication, were retained. Ultimately, 24 representative texts were selected as the analytical corpus.

Based on the practical functions and production contexts of the texts, this study first classified the corpus into three categories: planning and construction texts, adaptive reuse and operation texts, and marketing and promotion texts. Subsequently, considering key practical transformations during the renewal and place branding process of the historic district, the texts were further divided into three stages: the early stage (planning and construction stage, 2007–2012), the middle stage (activation and utilization stage, October 2013–May 2021), and the late stage (marketing and promotion stage, June 2021–2024). The temporal boundaries of these stages were defined according to critical milestones in the district’s development rather than the exact publication years of individual documents. Therefore, the assigned stage of each document reflects the practical context represented by the document rather than its publication year. Some documents published before or around the transition points were assigned to specific stages because they reflected transitional practices associated with the corresponding development processes. This stage division was not based on equal-length temporal intervals or derived retrospectively from textual results; rather, it was determined according to critical practical milestones in the district’s development, including changes in spatial construction, operational models, and dominant actors. These three stages reflect shifts in actors, practical objectives, and modes of textual production throughout the place branding process, while also corresponding to the transformation of physical spatial functions, as illustrated in Fig 3. During the planning and construction stage, efforts primarily focused on historic building preservation, streetscape façade restoration, and courtyard space renovation (Fig 3a–3c). During the activation and utilization stage, these spatial elements were further integrated with commercial, cultural, and tourism functions (Fig 3d–3f). During the marketing and promotion stage, streets, courtyards, historic buildings, cultural facilities, and consumption spaces were further reorganized into experiential settings and visual symbols for place branding communication and public representation (Fig 3g–3i).

**Fig 3:**
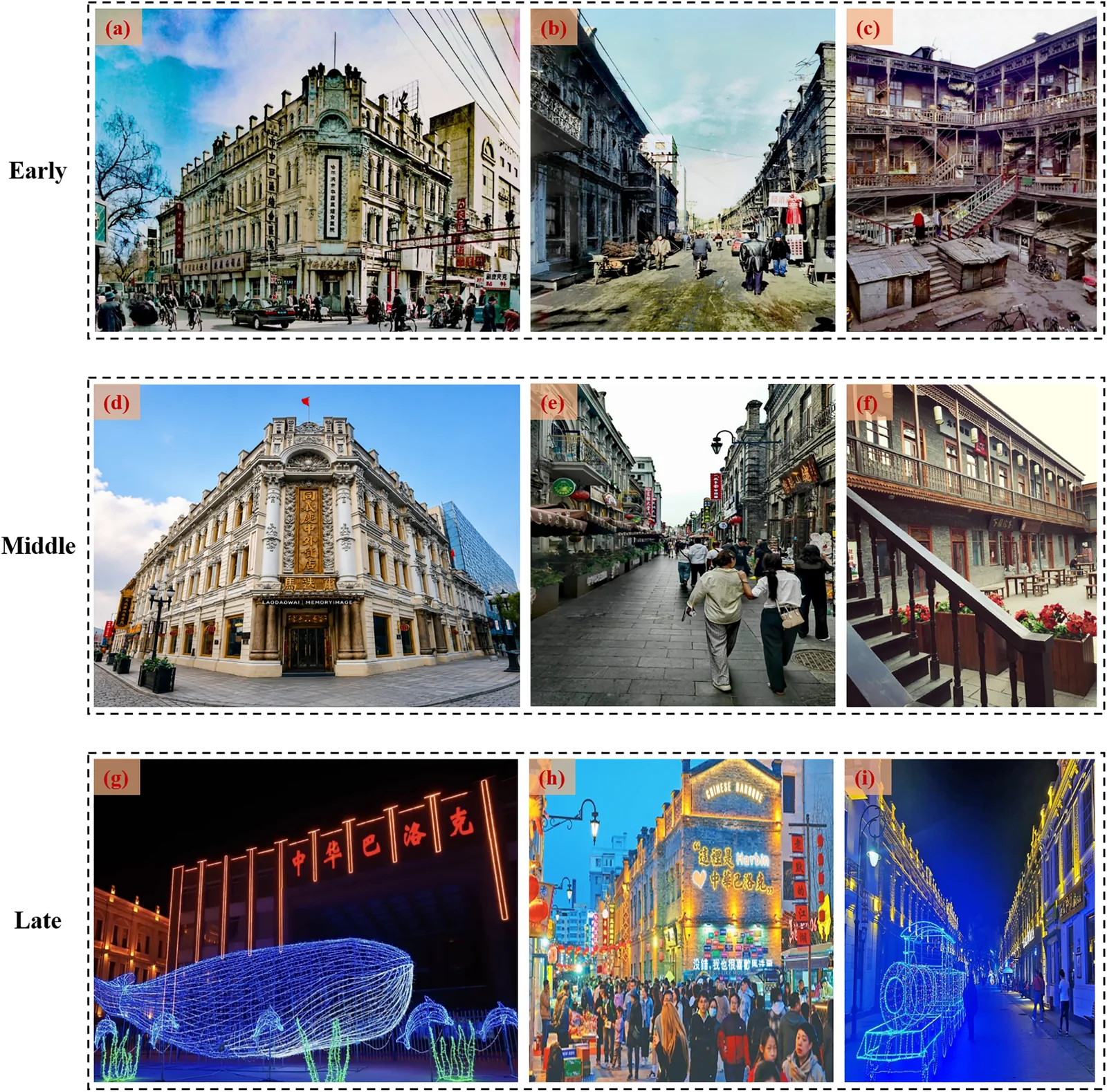
Representative spatial scenes of the Chinese Baroque Historic Area across the early stage (planning and construction stage), middle stage (activation and utilization stage), and late stage (marketing and promotion stage). Photographs in panels a, b, c, and e are reproduced with permission from Wenyong Song; photographs in panels d, f, g, h, and i are reproduced with permission from Songjiang Wang. All photographs are reproduced under a CC BY 4.0 license with permission from the respective copyright holders.

Specifically, the early stage (planning and construction stage) mainly focused on the identification of historical and cultural resources, spatial planning, and preservation-oriented renewal, comprising 5 texts. The middle stage (activation and utilization stage) corresponded to the transition from spatial construction to operational activation and began with the official opening and operation of the historic district in October 2013. During this stage, development priorities shifted toward commercial activation, functional transformation, and spatial operation, and ten texts were included. Although some documents included in this stage were published before October 2013 or were not continuously distributed throughout the entire period, they reflected preparatory activities, operational transitions, and functional transformation associated with the activation process. The late stage (marketing and promotion stage) was defined by the transfer of operational and management authority of the district to Longcai Technology Group in May 2021, after which the focus further shifted toward smart district development, brand communication, and the creation of consumption-oriented experiences, comprising 9 texts.

Although the number of texts varies across the three stages, this difference does not result from imbalanced sampling but reflects changes in text production mechanisms, participating actors, and practical demands at different stages. The absence of texts for certain years does not indicate a discontinuity in the development process; rather, it reflects variations in the intensity, purpose, and form of textual production during different stages. The planning and construction stage was primarily led by government departments and planning and construction institutions, and the corresponding texts mainly focused on spatial planning, construction implementation, and physical transformation. As the district entered the adaptive reuse and marketing and promotion stages, the number of participating actors gradually increased, while operational management, commercial investment promotion, cultural display, and branding activities expanded, resulting in a greater diversity of textual materials.

This study does not consider differences in text types as direct causes of changes in place identity narratives. Instead, such differences are understood as manifestations of varying practical objectives and action logics across different stages. The focus of this study is the structure of place identity narratives and their evolutionary processes as jointly represented across multiple types of texts.

To improve the ability of the LDA topic model to identify the evolutionary characteristics of place identity narratives, reduce textual noise, and retain information related to historical representation, spatial meanings, cultural symbols, and place identity construction, this study first conducted systematic preprocessing of the textual corpus. The preprocessing procedures mainly included text format standardization, manual screening, Chinese word segmentation, stop-word removal, and vocabulary construction. First, all collected texts were manually reviewed to remove duplicated materials, irrelevant information, and texts lacking substantive narratives related to spatial, cultural, operational, or branding practices. Subsequently, Chinese word segmentation was performed using the Jieba segmentation tool (version 0.42.1) implemented in the Python 3.11 environment.

To improve topic identification performance, this study constructed a customized stop-word list based on commonly used Chinese stop-word resources and the linguistic characteristics of planning, policy, operational, and promotional texts. Specifically, procedural and governance-related expressions, temporal and process-oriented terms, highly abstract general expressions, and entity terms directly referring to the research object or administrative spaces were removed to reduce textual noise and avoid generating spurious topics dominated by place names or administrative terminology. For example, policy-oriented expressions such as “推进”, “加强”, “实施” and “落实” as well as general descriptive terms such as “方面”, “情况” and “内容” were removed. In addition, terms with limited discriminatory power for thematic differentiation, including “region”, “project”, and “function”, were excluded. Meanwhile, words associated with historical and cultural representations, spatial experiences, everyday life, local memories, and cultural symbols were retained to ensure that the model could capture the information characteristics and structural differences of place identity narratives across different development stages. The basic characteristics of the corpus before and after preprocessing, including the number of texts, original text size, number of segmented words, number of valid words, and vocabulary size, are presented in Table 1.

**Table 1:** Corpus statistics and text-level analytical units.

Item	Value
Number of textual documents	24
Raw corpus size (Chinese characters only)	91,833
Raw corpus size after initial segmentation (words)	48,813
Tokens after preprocessing	16,498
Vocabulary size after preprocessing	3,177

After completing text preprocessing, this study transformed the corpus into a term frequency-based document–term matrix, which was used as the input for the LDA topic model. The LDA model was implemented using the Latent Dirichlet Allocation module in scikit-learn under the Python 3.11 environment, and model parameters were estimated using the variational Bayesian inference approach. The main model settings and hyperparameter configurations are presented in Table 2, including the maximum number of iterations, document–topic prior parameter (α), topic–word prior parameter (β), random seed, learning method, and learning offset parameter.

**Table 2:** Main LDA implementation settings and hyperparameter configuration.

Parameter	Symbol/ scikit-learn setting	Value
Maximum iterations	max_iter	50
Document-topic prior	α (doc_topic_prior)	0.1
Topic-word prior	β (topic_word_prior)	0.01
Random seed	random_state	42
Learning method	learning_method	batch
Learning offset	learning_offset	50

To determine an appropriate number of topics, this study compared LDA models with different numbers of topics by considering both model fitting performance and topic interpretability. Specifically, perplexity and topic coherence were employed to evaluate model performance under different topic numbers. Perplexity measures the model’s ability to fit the distribution of textual data, with lower values generally indicating better predictive performance. Topic coherence evaluates the semantic associations among high-frequency words within each topic, with higher values generally indicating greater interpretability. Since the lowest perplexity value does not necessarily correspond to the most theoretically meaningful topic structure, this study integrated the results of both evaluation metrics and further determined the optimal number of topics based on the theoretical background of the research context and the semantic interpretability of topics, thereby improving the stability and interpretability of topic identification results.

After determining the optimal number of topics, this study interpreted and labeled topic semantic characteristics based on pyLDAvis visualization results, topic–word distributions, and topic-related word clouds. Meanwhile, the LDA model generated a document–topic probability matrix (document-topic distribution), from which topic weights across different stages were further calculated and used as the basis for subsequent information structure analysis.

### 3.3 Information structure analysis

Based on the topic probability distributions obtained from the LDA model, this study further conducts an information structure analysis of place identity narratives. To clarify the concept of information structure used in the subsequent analysis, this study conceptualizes place identity narratives as a dynamic information structure composed of multiple cultural information elements, based on the theoretical understanding that local cultural elements are continuously selected, reinforced, and reorganized throughout the place branding process. This information structure not only reflects the relative importance of different topics at specific stages but also captures the degree of structural concentration among topics and the processes of information adjustment across different development stages. Based on three analytical dimensions—information importance, structural concentration, and stage-based variation—this study develops an information structure analysis framework consisting of four indicators: information priority, structural dominance, information dispersion, and information compression (Table 3). Specifically, information priority measures the topic weight of a specific theme within the text corpus of a given stage and its relative importance in the overall narrative structure. Structural dominance measures the extent to which information resources are concentrated on dominant topics, reflecting the degree of concentration in topic weight distributions within stage-specific narrative structures. Information dispersion measures the magnitude of changes in the weight of a specific topic across different development stages. Information compression, based on the entropy of topic weight distributions, evaluates the overall degree of concentration of place identity narrative structures within a specific stage.

**Table 3:** Analytical indicators of information structure and their operational definitions.

Concept	The operational implications in this study
Information priority	The thematic weight of a given information theme within a text collection at a specific stage is used to measure its relative importance within the overall information structure.
Structural dominance	The concentration degree of thematic weight distribution reflects the extent to which information resources are concentrated among dominant themes. Higher values indicate stronger dominance of a limited number of themes within the information structure.
Information dispersion	The range of thematic weights across different stages measures the extent of variation in the narrative position of a specific theme over time. Higher values indicate greater cross-stage shifts in thematic importance.
Information compression	Based on normalized Shannon entropy, information compression measures the concentration degree of thematic weight distribution within a specific stage. Higher values indicate a more concentrated information structure, whereas lower values indicate a more diversified thematic distribution.

Before calculating the proposed indicators, this study first derived stage-level topic weights from the document–topic probability matrix generated by the LDA model. Let *θ*_*dk*_ denote the probability of topic k in document d, and let $n_{s}$ represent the number of texts included in stage s. The average weight of topic k within stage s is calculated as shown in Equation (1).


$$\begin{array}{c} S_{sk}=\frac{\text{1}}{n_{s}}\sum_{d\in s}\theta_{dk} \end{array}$$ (1)

Because the sum of topic probabilities for each document equals one, the following constraint is satisfied for each stage, as expressed in Equation (2).


$$\begin{array}{c} \sum_{k=\text{1}}^{K}S_{sk}=\text{1} \end{array}$$ (2)

Where K represents the number of topics identified by the LDA model. Based on the stage-level topic weights, this study further constructs four information structure indicators to characterize the importance, concentration, and dynamic variation of place identity narratives across different stages.

First, to measure the relative importance of a specific topic within the place identity narrative structure of a given stage, this study uses the stage-level topic weight as the measurement indicator for information priority, as shown in Equation (3).


$$\begin{array}{c} IP_{sk}=S_{sk} \end{array}$$ (3)

Where $IP_{sk}$ represents the information priority of topic k in stage s. A higher value indicates that the topic has greater relative importance and a more prominent position within the narrative structure of that stage.

Second, to measure the concentration of topic weights within each stage, this study adopts structural dominance based on the Herfindahl-type sum-of-squares index, as shown in Equation (4).


$$\begin{array}{c} SD_{s}=\sum_{k=\text{1}}^{K}S_{sk}^{\text{2}} \end{array}$$ (4)

Where $SD_{s}$ represents the structural dominance of stage s, with a theoretical range of [1/K, 1]. When topic weights are evenly distributed across all topics, $SD_{s}\;$ approaches its minimum value of 1/K. When narrative resources become concentrated among a small number of dominant topics, the value increases. When the narrative structure is highly concentrated on a single topic, $SD_{s}$ approaches 1. Therefore, a higher value of this indicator indicates that place identity narratives are more concentrated around a limited number of dominant topics.

Third, to identify the extent to which the importance of a specific topic changes across different stages, this study calculates information dispersion based on the range of topic weights across stages, as shown in Equation (5).


$$\begin{array}{c} D_{k}=max\left(S_{sk}\right)-min\left(S_{sk}\right) \end{array}$$ (5)

Where $D_{k}$ represents the information dispersion of topic k. A higher value of this indicator indicates that the topic exhibits greater variation in weight across different stages and undergoes more substantial adjustments in its narrative position.

Fourth, to measure the concentration of stage-level topic structures from the perspective of overall distribution, this study constructs information compression based on Shannon entropy. First, the entropy of the topic distribution within each stage is calculated, as shown in Equation (6).


$$\begin{array}{c} H_{s}=-\sum_{k=\text{1}}^{K}S_{sk}lnS_{sk} \end{array}$$ (6)

The information compression index is then obtained through further normalization, as shown in Equation (7).


$$\begin{array}{c} IC_{s}=\text{1}-\frac{H_{s}}{lnK} \end{array}$$ (7)

Where $IC_{s}$ represents the information compression of stage s. When topic weights are completely evenly distributed, the entropy reaches its maximum value and $IC_{s}\;$approaches 0. When topic weights become concentrated on a small number of topics, entropy decreases and $IC_{s}$ increases. Therefore, a higher value of information compression indicates that the narrative structure of the corresponding stage is more concentrated.

To clarify the measurement relationships among the proposed indicators, this study further distinguishes their respective units of observation and analytical functions. Information priority takes the “stage–topic” pair as the unit of observation and measures the relative importance of topics within different stages. Information dispersion takes the “topic” as the unit of observation and captures the magnitude of changes in topic weights across stages. Structural dominance and information compression take the “stage” as the unit of observation and describe the overall concentration of information structures. Together, the four indicators characterize the organizational features of place identity narrative information structures from three dimensions: topic importance, stage-based variation, and structural concentration. Specifically, structural dominance is calculated based on the sum of squared topic weights and emphasizes the concentration of dominant topics, whereas information compression is derived from Shannon entropy and reflects the overall balance of topic distributions. Although these two indicators are theoretically related, they capture different structural characteristics by emphasizing local dominance and overall distribution patterns, respectively.

To further examine whether potential redundancy existed among the indicators within the proposed indicator system, this study conducted Pearson correlation analysis based on the calculated topic weights across different stages. The results revealed differentiated correlation patterns among the indicators. Specifically, structural dominance exhibited a strong positive correlation with information compression (r=0.999, p<0.001), which is consistent with the theoretical expectation that both indicators characterize the concentration of information structures. Although both indicators reflect structural concentration, structural dominance captures the concentration of topic weights, whereas information compression reflects the overall distributional characteristics of the information structure, indicating different analytical emphases. In addition, information priority showed a moderate positive correlation with information dispersion (r=0.658, p=0.001), suggesting that topics with higher weights tended to experience more pronounced adjustments across different stages. Except for these relationships, no other indicator combinations exhibited significant correlations (p>0.05). These results demonstrate that the proposed indicators provide complementary perspectives for characterizing the evolution of place identity narrative information structures, including topic importance, structural concentration, and the magnitude of stage-based changes.

Furthermore, to determine whether changes in topic weights across different stages were statistically significant, this study conducted nonparametric tests based on document-level topic probability distributions generated by the LDA model. Because the number of texts varied across stages and the topic probability data did not satisfy the assumptions of normality and homogeneity of variance, the Kruskal–Wallis H test was employed to compare differences in the distributions of document-level topic weights among different stages. For topic k, the test statistic is calculated as shown in Equation (8).


$$\begin{array}{c} H_{k}=\frac{\text{1}\text{2}}{N(N+\text{1})}\sum_{i=\text{1}}^{g}\frac{R_{ik}^{\text{2}}}{n_{i}}-\text{3}(N+\text{1}) \end{array}$$ (8)

Where N represents the total number of texts, g represents the number of stages, $n_{i}$ represents the number of texts included in stage i, and *R*_*ik*_ represents the rank sum of topic k in stage i. Ties in ranking were adjusted during the calculation. Because multiple topics were independently tested, the Benjamini–Hochberg procedure was further applied to control the false discovery rate (FDR) and reduce the risk of Type I errors caused by multiple comparisons. The adjusted p-values were used as the final criteria for determining statistical significance.

Additionally, to enhance the interpretability of the topic analysis results, this study incorporates keyword co-occurrence network analysis to examine the relationships among different topics and further discusses the organizational characteristics of the information structure underlying place identity narratives from a relational perspective.

## 4. Results

### 4.1 Topic synthesis of the Chinese Baroque identity narrative

The number of topics is one of the key parameters that substantially influences the effectiveness of the LDA topic model in identifying the latent thematic structure within text [43]. An insufficient number of topics may lead to excessive consolidation of distinct semantic dimensions, thereby weakening the model’s ability to distinguish heterogeneous textual information. Conversely, an excessive number of themes tends to cause semantic fragmentation and high overlap among themes, consequently reducing the stability and interpretability of model results.

To determine an appropriate number of topics, this study employed a comprehensive evaluation approach integrating perplexity and topic coherence. Perplexity was used to assess the ability of the LDA model to fit the distribution of textual data, whereas topic coherence was used to evaluate the semantic associations among high-probability words within each topic and the interpretability of the resulting topics. The performance of LDA models with different numbers of topics (K) was further compared, and the results are presented in Fig 4. As the number of topics increased, the overall perplexity of the model showed a decreasing trend. When K increased from 1 to 7, perplexity declined substantially; however, when K exceeded 7, the variation in perplexity became relatively stable, indicating that further increases in the number of topics provided limited improvement in the model’s fitting performance. Meanwhile, the topic coherence results indicated that the semantic consistency of the model varied across different values of K, with K=7 achieving a relatively high level of coherence. Therefore, by jointly considering the results of perplexity and topic coherence, this study determined K=7 as the optimal number of topics.

**Fig 4:**
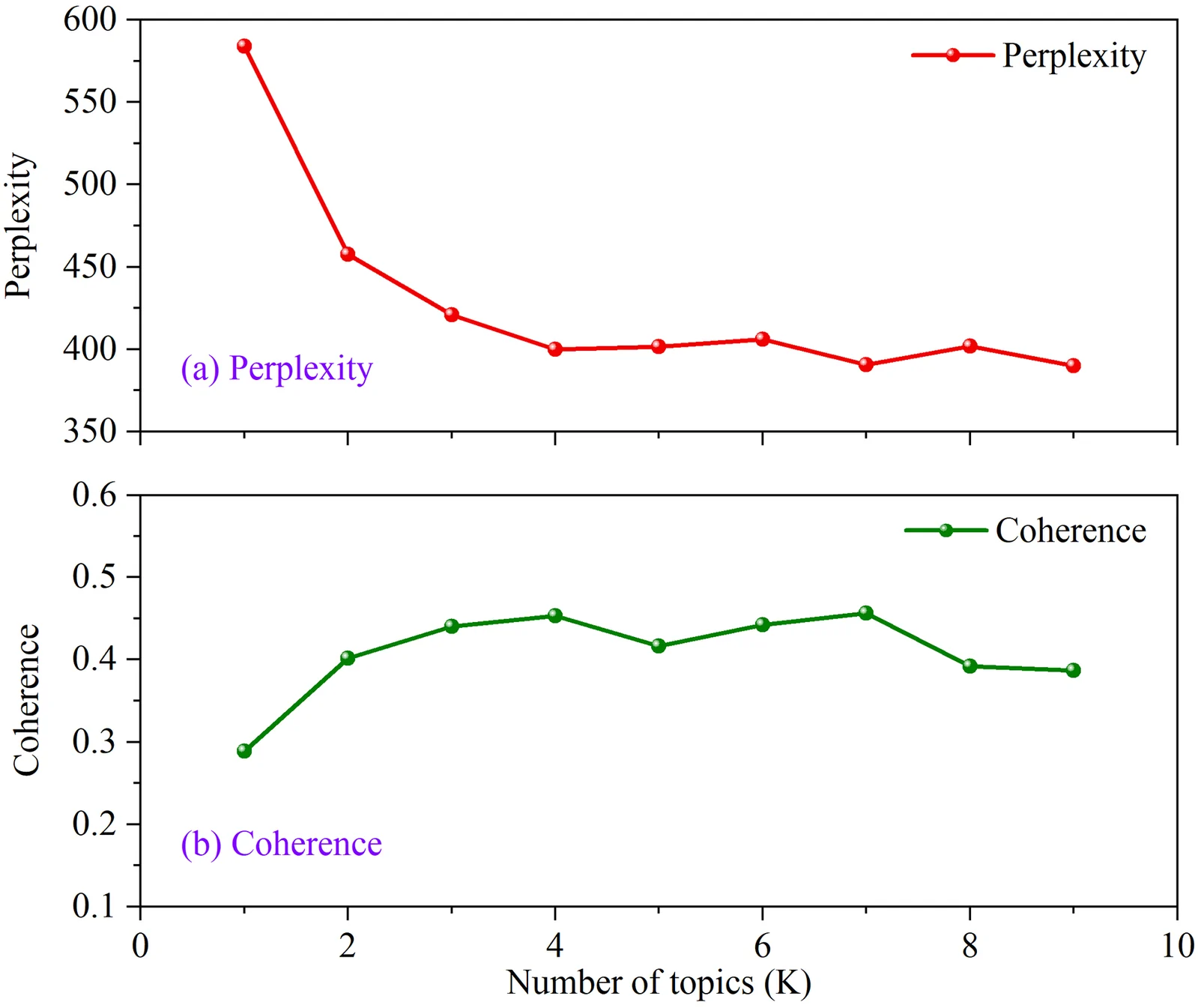
Perplexity and coherence across different numbers of topics.

To investigate the degree of differentiation and semantic independence among themes when the number of themes is set to 7, this study further employed pyLDAvis to examine the LDA thematic model results visually [44]. The output is shown in Fig 5. Each theme represents distinct concepts and perspectives within the corpus, visualized as a single circle to highlight both its prevalence and uniqueness across documents. The distance between circle centers indicates the degree of similarity among themes. In contrast, each theme’s area reflects the “relevance score” calculated by pyLDAvis—larger areas indicate greater importance or prominence within the documents. Overlapping circles indicate shared characteristic words among different themes. Since LDA themes are generated from the included text, some themes exhibit partial overlap in scope, consistent with the inherent characteristics of the LDA thematic modeling method. When K=7, the circles representing each topic are distributed relatively evenly across different quadrants without substantial overlap, maintaining a certain distance in the two-dimensional space. This indicates that the topics are well distinguished and have strong separability in the semantic space. Therefore, by integrating the results of perplexity, topic coherence, and topic visualization analyses, this study determined K=7 as the optimal number of topics. The selected model achieved a satisfactory balance between model fit, topic coherence, and topic distinctiveness, providing reliable and interpretable topic structures for subsequent analysis.

**Fig 5:**
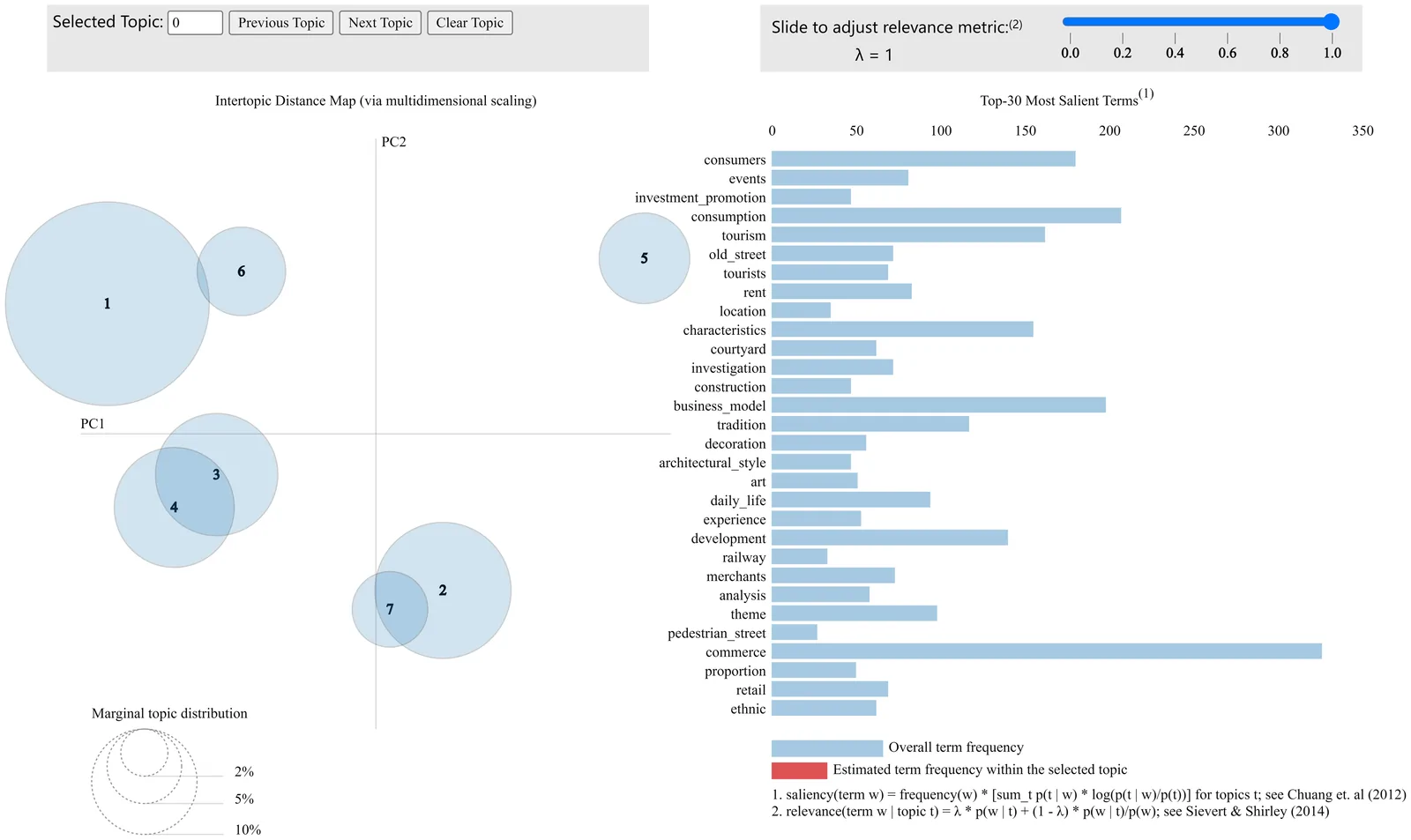
The layout of LDAvis, with the intertopic distance map (via multidimensional scaling) for the seven identified topics on the left and the Top-30 salient terms (via relevance metric λ) of the selected topic on the right. Linked selections allow compact exploration of topic–term relationships.

To reveal the distribution characteristics of themes and their associated keywords within the Chinese Baroque identity narrative texts, this study statistically analyzed and presented the distribution patterns of each identified theme and its high-probability core feature words. The results are shown in Table 4. These seven themes encompass multiple information dimensions, including commercial operations, tourism experiences, historical architectural imagery, spatial planning, daily life, and brand promotion. Overall, these themes do not form mutually exclusive narrative units but collectively constitute an internally interconnected textual information structure. Keyword distribution reveals substantial lexical overlap across themes. Spatial and historical imagery such as “old streets”, “Laodaowai”, “century-old”, “tradition”, and “architectural style” recur across multiple themes, forming foundational elements within place identity narratives. This indicates that place identity narratives do not revolve around a single theme but center on relatively stable core imagery that is continuously invoked across different textual contexts. Simultaneously, lexical overlap does not imply thematic homogeneity. The same core imagery forms differentiated combinations with terms related to commercial operations, planning and construction, tourism experiences, and daily life across different themes, reflecting their varying informational functions and narrative orientations in texts from different stages. Thus, the place identity narrative of Chinese Baroque exhibits a non-linear evolution in its information structure. While core imagery remains relatively stable, its informational priority and structural dominance undergo phased adjustments amid the coexistence and reorganization of multiple themes. This ultimately drives the overall information structure toward gradual compression and stabilization.

**Table 4:** Distribution of topics and their high-probability keywords.

ID	Topic category	Top 10 high-probability terms
Topic_0	Commercial consumption and business operations	Commerce, consumption, consumers, business_model, entertainment, catering, rent, investigation, retail, merchants
Topic_1	Tourism-oriented commercialization and cultural experience	Tourism, development, commerce, characteristics, theme, catering, tradition, experience, commercial_street, ethnic
Topic_2	Traditional courtyards and historic architectural imagery	Courtyard, tradition, restaurant, century, shop_sign, manage, decoration, commerce, Chuangguandong, architectural_style
Topic_3	Artistic intervention and image modernization	Tourists, art, architectural_style, decoration, construction, railway, experience, corporate_group, tradition, technology
Topic_4	Spatial planning and public environment construction	Characteristics, planning, tradition, square, engineering, space, tourism, commerce, landscape, daily_life
Topic_5	Everyday life and vernacular urban culture	Old_street, location, daily_life, tradition, everyday_life, coffee, international, steamed_buns, courtyard, relief
Topic_6	Branding, promotion, and investment attraction	Events, investment_promotion, old_street, pedestrian_street, commerce, image, tourists, Baidu, brand, characteristics

To visually represent the distribution characteristics and clustering patterns of themes, this study employs word clouds as an auxiliary analytical tool, using word frequency to convey the importance of keywords across themes [45]. The word cloud of thematic keywords for the identity narrative of the Chinese Baroque Historic Area is shown in Fig 6. Different themes exhibit distinct differences in keyword composition and semantic orientation. Overall, keyword distributions within each theme display distinct clustering patterns, with a few high-frequency terms visually dominating the space. This indicates that distinct thematic information units possess relatively stable semantic cores and informational boundaries within the textual system. Examining specific thematic characteristics: Some themes center on terms like “old streets”, “courtyard compounds”, “century”, “tradition”, and “architectural style”, with keywords primarily pointing to historical spatial forms, traditional architectural imagery, and local cultural memory. This reflects a focus in related texts on interpreting and defining historical landscapes and spatial features during specific phases. Concurrently, other themes center on terms such as “consumption”, “tourism”, “visitors”, “activities”, “investment promotion”, “branding”, and “experience”, emphasizing commercial operations, tourism development, and communication practices. This shift demonstrates how subsequent texts progressively intensify their focus on consumption scenarios, experiential design, and image cultivation.

**Fig 6:**
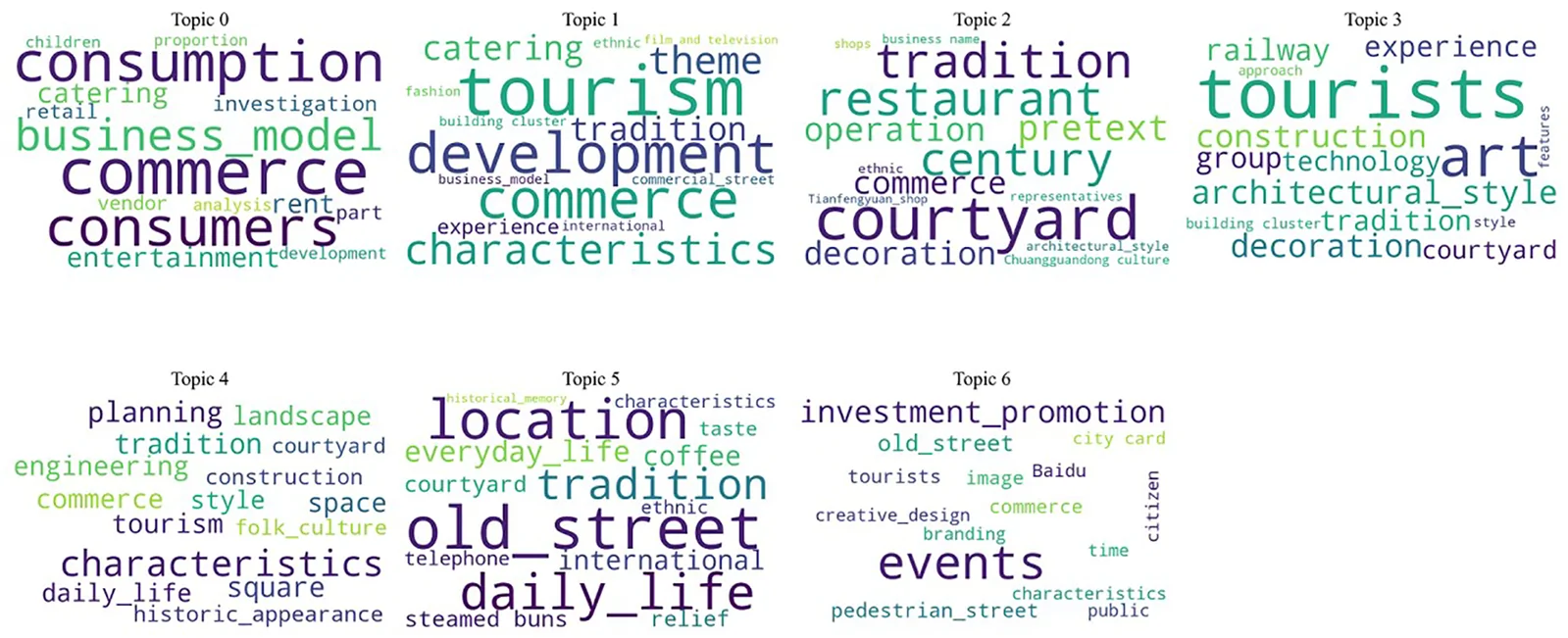
The word cloud of topic-relevant words.

It is noteworthy that while themes vary in their core keywords, terms such as “tradition”, “commerce”, “tourism”, and “old streets” recur across multiple themes, forming distinct combinations within different semantic clusters. This phenomenon indicates that core spatial elements and historical imagery maintain relative stability within the long-term textual system. Meanwhile, their informational functions and narrative orientations are adjusted across different textual phases.

### 4.2 The evolution of information priority and structural dominance in the narrative of Chinese Baroque identity

To analyze the structural positioning of different thematic information elements within long-term textual systems and how this positioning evolves as texts progress through their developmental stages, this study introduces two metrics: information priority and structural dominance. By comparing the relative importance and structural distribution characteristics of each theme across early, middle, and late-stage texts, the research aims to reveal the evolutionary process of information structure—from concentration to differentiation and ultimately to reorganization—across textual stages. The overall thematic weight distribution of the Chinese Baroque identity narrative texts is presented in Table 5.

**Table 5:** Overall topic weights in the Chinese Baroque identity narrative texts.

ID	Weight (%)
Topic_0	4.23
Topic_1	17.27
Topic_2	8.78
Topic_3	13.78
Topic_4	37.10
Topic_5	9.46
Topic_6	9.37

From a holistic perspective, the distribution of themes within the Chinese Baroque identity narrative text shows a pronounced imbalance. Topic_4 has the highest overall weight (37.10%) and occupies a dominant position within the overall information structure. Following this are Topic_1 (17.27%) and Topic_3 (13.78%), which collectively form the secondary core themes within the textual system. In contrast, Topic_0 exhibits the lowest overall weight (4.23%), while the remaining topics show relatively dispersed weight distributions. This indicates substantial disparities in the structural positioning of different information units within the long-term textual system.

To further investigate the relative importance and distribution characteristics of various themes within the overall information structure across different stages of texts, a comparative analysis of the average weight of each theme in early, middle, and late-stage texts was conducted. This aims to reveal the evolutionary process of information structure—from concentration to differentiation and then to reorganization—across textual practices spanning these stages. The distribution of topic weights and statistical results are presented in Table 6.

**Table 6:** Stage-based topic weights and statistical differences across different phases.

ID	Early (%)	Middle (%)	Late (%)	Kruskal–Wallis H	Raw p-value	FDR-adjusted p-value
Topic_0	0.11	10.07	0.03	6.35	0.04	0.15
Topic_1	0.11	30.42	12.19	1.77	0.41	0.50
Topic_2	0.11	10.07	12.16	1.71	0.43	0.50
Topic_3	19.90	0.07	25.61	2.82	0.24	0.43
Topic_4	79.55	41.70	8.42	7.84	0.02	0.14
Topic_5	0.11	6.56	17.88	1.12	0.57	0.57
Topic_6	0.11	1.11	23.71	3.56	0.17	0.39

The information priority and structural dominance of various topics underwent substantial adjustments across different textual phases, exhibiting distinct stage-specific characteristics. To further examine whether variations in topic weights across different stages represented statistically significant differences, a Kruskal–Wallis H test was conducted based on document-level topic weights. The results showed that Topic_0 (H=6.35, raw p=0.04) and Topic_4 (H=7.84, raw p=0.02) exhibited nominal differences before multiple comparison correction. However, after Benjamini–Hochberg false discovery rate (FDR) adjustment, none of the topics showed statistically significant differences (adjusted p>0.05). Therefore, the observed variations in topic weights were not interpreted as statistically confirmed stage differences, but rather as manifestations of the continuous adjustment of thematic priorities, structural relationships, and narrative functions within the long-term textual information system. Subsequent analyses focus on information structural evolution by examining topic weight distributions, structural concentration, cross-stage variation, and semantic characteristics of themes.

In early-stage texts, information structure was highly concentrated, with Topic_4 holding an average weight of 79.55%. Together with Topic_3 (19.90%), these two topics accounted for nearly the entire information space, while the weights of the other topics approached zero. This indicates that during the planning and construction stage, place identity narratives were primarily centered on spatial planning, traditional aesthetics, and institutional definitions, resulting in an information structure characterized by clear single-topic dominance. Specifically, Topic_4 represents core practices related to historical spatial preservation, architectural character shaping, and environmental reconstruction, including the conservation of traditional buildings, the restoration of street patterns, and the reshaping of courtyard spaces. For example, the section from Nantoudao Street to Nansidao Street focused on preserving and restoring the street-facing facades of Chinese Baroque buildings; the section from Jingyang Street to Nantoudao Street reproduced historical cultural symbols through newly constructed and historically styled architectural nodes; and the section from Nansandao Street to Nansidao Street strengthened representations of traditional living scenes through courtyard space reconstruction. Therefore, the prominence of the spatial planning topic during the planning and construction stage demonstrates that place identity narratives were gradually constructed through material spatial interventions and the reproduction of historical symbols. This finding is also supported by the high structural dominance (0.67) and information compression (0.72) values reported in the information structure analysis, suggesting that early-stage narratives were strongly concentrated around a limited number of cultural elements.

Although Topic_4’s weight decreased to 41.70%, it maintained relative dominance; simultaneously, the weights of Topic_1 (30.42%) and Topic_0/Topic_2 (both 10.07%) increased substantially, indicating that information themes such as commercial development, tourism experiences, and functional positioning were gradually integrated into the core structure. This redistribution of topic weights reflects a shift in the focus of place identity narratives from spatial construction toward the integrated development of cultural experiences, commercial functions, and tourism-oriented consumption spaces. This transformation corresponds to the changing patterns of spatial utilization after the district was officially opened and operated. For example, in the area from Nan Erdao Street to Nantoudao Street, the operating entity created emotionally engaging street experiences by incorporating historical memories and local cultural narratives. In the area from Nansidao Street to Nansandao Street, landscape nodes such as the “Chinese Baroque Back Garden” were created by integrating traditional cultural elements, enabling historical buildings and street spaces to gradually transform from objects of preservation into carriers of cultural experiences and consumption activities. Therefore, the evolution of the information structure during the activation and utilization stage demonstrates that place identity narratives expanded from a focus on “preservation and representation” toward “experience and utilization”. Although Topic_0 exhibited a relatively high H statistic (H=6.355), its FDR-adjusted p-value did not reach statistical significance (adjusted p=0.15). Therefore, the change in topic weight during this stage should be interpreted as a redistribution of narrative emphasis rather than a statistically significant stage transition. Compared with the planning and construction stage, topic weights became more dispersed, and the decline in structural dominance to 0.29 indicates that place identity narratives gradually shifted from being dominated by a single core theme toward a structure in which multiple information elements jointly contributed to narrative construction.

In the late texts, the information structure undergoes further reorganization and tends toward relative equilibrium. Topic_3 (25.61%), Topic_6 (23.71%), and Topic_5 (17.88%) emerge as new high-weight themes, while Topic_4—which previously held dominant status—sees its weight substantially decline to 8.42%. This transformation indicates that the core content of place identity narratives gradually shifted from spatial planning and architectural preservation toward brand communication, consumption experiences, and representations of everyday life. The textual focus moved from the construction of material spaces to the organization of information centered on communication practices and experiential consumption. Compared with the earlier emphasis on historical spaces themselves, this stage placed greater attention on how brand promotion, consumption-oriented scenarios, and everyday experiences could reinforce the image of the historic district. This thematic transformation corresponded with the continuous renewal of commercial spaces and business formats within the district. For example, the area from Nan Erdao Street to Nansandao Street introduced experiential businesses, including cafés, music bars, travel photography studios, and museums, while the area from Jingyang Street to Nantoudao Street developed consumption spaces featuring creative dining and cultural performances. These transformations gradually promoted the district’s transition from a historical display space into a cultural consumption destination. Therefore, the restructuring of the information structure during the marketing and promotion stage demonstrates a further expansion of the functions of place identity narratives, whereby brand communication, consumption scenario creation, and everyday life experiences collectively contributed to the continued dissemination and reinforcement of the district’s image. Although Topic_4 exhibited the highest H statistic (H=7.84, raw p=0.02), its FDR-adjusted result remained statistically non-significant (adjusted p=0.14). Therefore, the decline in Topic_4 weight should be interpreted as a structural adjustment in information priority rather than a statistically validated stage difference. The further decrease in structural dominance to 0.19 indicates that place identity narratives no longer relied on a single dominant theme, but instead developed a relatively balanced information structure composed of multiple cultural, commercial, and experiential elements.

Overall, although the stage differences of individual topics did not remain statistically significant after FDR correction, the combined changes in topic weight distributions, structural concentration, and corresponding spatial practices reveal the evolutionary process of place identity narrative information structures. During the early stage (planning and construction stage), place identity was primarily shaped through architectural preservation and spatial reconstruction to establish a historical and cultural image. During the middle stage (activation and utilization stage), cultural landscape creation and functional transformation further endowed spaces with experiential value. During the late stage (marketing and promotion stage), brand communication, the introduction of commercial activities, and the creation of consumption-oriented scenarios strengthened the dissemination of the district’s image. Therefore, place identity narratives should not be understood as fixed expressions pre-existing within texts; rather, they represent evolving information structures continuously adjusted and constructed through the interaction of text production, spatial transformation, and operational practices.

### 4.3 Thematic fluctuations and functional evolution in the narrative of Chinese Baroque identity

To further analyze the degree of dispersion and structural adjustment characteristics of different themes during cross-stage evolution, this study compares the variance, standard deviation, and range of theme weight fluctuations across early, middle, and late-stage texts from the perspective of theme weight fluctuation amplitude and structural stability. The weight fluctuations of each theme across different stages are shown in Table 7.

**Table 7:** Cross-stage variability of topic salience.

ID	Variance (percentage points²)	Standard Deviation (percentage points)	Range (percentage points)
Topic_0	33.34	5.77	10.04
Topic_1	232.74	15.26	30.30
Topic_2	41.43	6.44	12.04
Topic_3	179.64	13.40	25.54
Topic_4	1266.54	35.59	71.13
Topic_5	80.94	9.00	17.77
Topic_6	178.14	13.35	23.60

Overall, different topics exhibit considerable variations in structural stability, indicating distinct patterns of weight fluctuation across textual phases. Among them, Topic_4 demonstrates the most pronounced volatility across all three metrics, with variance (1266.54), standard deviation (35.59), and range (71.13) substantially exceeding those of other topics. This result indicates that Topic_4’s information priority undergoes substantial changes across different phases, exhibiting high phase sensitivity. In contrast, Topic_0 and Topic_2 exhibit relatively low variance, standard deviation, and range, suggesting limited fluctuations in their weights across phases. Such topics maintain relatively stable yet non-dominant structural positions within long-term textual systems, tending to function as persistent foundational or supplementary information units that provide stable support to the overall information structure. Topics_1, Topic_3, and Topic_6 exhibit moderately high structural volatility, with variances and ranges considerably higher than those of low-volatility topics but lower than those of Topic_4. This characteristic indicates that these topics exhibit strong adaptability across different phases, often being substantially amplified or diminished during specific periods and showing relatively high but non-extreme cross-phase fluctuations. Topic_5’s fluctuation level is intermediate, showing gradual strengthening in late phases but overall relatively smooth changes.

To further elucidate the differing roles of place identity narrative texts across distinct phases, this study analyzes the functional positioning and role division of early, middle, and late-stage texts within the overall information system. The primary informational functions undertaken by different text types in each phase are presented in Table 8.

**Table 8:** Dominant characteristics and information functions of narrative texts across stages.

Stage	Dominant text type	Salience pattern	Dominant themes	Primary information function
Early	Planning & policy texts	Extreme concentration	Topic_4	Structural definition & narrative framing
Middle	News & promotional texts	Multi-theme coexistence	Topic_4 & Topic_1	Information translation & thematic expansion
Late	Promotional & new media texts	Relative equilibrium	Topic_3, Topic_6 & Topic_5	Narrative consolidation & Place branding

As a continuously generated textual information system, texts at different stages not only exhibit variations in thematic weight and structural dominance but also undergo shifts in their core functions within the overall information system. In the early phase, texts primarily consist of planning documents and policy texts, characterized by highly concentrated information priorities, with a single or a few themes dominating.

In the early phase, texts primarily consist of planning documents and policy texts, and the information structure is characterized by extreme concentration. Topic_4 accounts for 79.55% of the thematic weight, with a high structural dominance (0.67) and information compression (0.72), indicating that place identity narratives at this stage were mainly organized around a limited number of core cultural elements. The relatively high information compression further indicates that diverse local cultural meanings were compressed and concentrated into a limited set of spatial and historical symbols during this stage. Meanwhile, Topic_4 exhibited the highest information dispersion among all topics, reaching 71.13%, suggesting that spatial planning-related narratives were not only the informational core during the early stage but also the thematic element experiencing the greatest degree of adjustment throughout the subsequent restructuring of place identity narrative structures. The primary function of texts at this stage is to define and institutionally frame the research subject structurally. By clarifying spatial boundaries, functional positioning, and conservation principles, they provide a stable structural framework for subsequent narratives. At this point, the text system emphasizes “what defines it” and “how it should be understood”, with identity narratives serving primarily normative and framing functions. Rather than presenting diverse cultural meanings, early-stage textual practices primarily established the basic interpretive framework through the selection and reinforcement of specific cultural elements.

Entering the middle stage, news reports and promotional texts become heavily integrated, substantially increasing the coexistence of multiple themes within the text system. Although Topic_4 remains the dominant theme with a weight of 41.70%, other themes begin to participate in meaning production, and the cumulative weight of the top two themes reaches 72.12%. Meanwhile, structural dominance declines to 0.29, suggesting a transition from a highly concentrated narrative structure toward a multi-theme configuration. Information priority shifts from high concentration to relative dispersion. Different themes are invoked in parallel during this phase, forming connections between planning discourse and communication discourse. This transformation is also reflected in the increased information dispersion of several themes. Specifically, Topic_1 (30.30%), Topic_3 (25.54%), and Topic_6 (23.60%) exhibited relatively high levels of information dispersion, indicating that themes related to commercial development, experiential communication, and cultural expression possessed strong adaptive capacities throughout the cross-stage evolution process. These themes gradually became important components of local meaning production as the place branding process progressed. Accordingly, the core function of identity narratives shifts from pure structural definition to information translation and meaning expansion: on one hand, transforming earlier institutional and planning information into more readable, communicable expressions, while, on the other hand, embedding the originally abstract planning framework within concrete narrative contexts by introducing commercial, tourism, and experiential elements. This facilitates the diffusion and comprehension of identity information across diverse audiences.

In the late stage, textual types further consolidated toward promotional materials and new media coverage, with thematic structures tending toward relative equilibrium. The dominance of a single theme further decreases, with the highest thematic weight declining to 25.61% and five themes exceeding 10% of the total thematic weight. The structural dominance (0.19) and information compression (0.12) reach their lowest levels across stages, indicating that place identity narratives are no longer organized around a small number of dominant themes but instead achieve a more balanced distribution among multiple information elements. The lower information compression indicates that place identity narratives in the later stage were no longer highly condensed around a single symbolic pathway, but instead relied on the integration of multiple information elements, including cultural, commercial, experiential, and everyday life dimensions, to sustain the overall place image. Meanwhile, information dispersion further reveals the differentiated roles of various topics during this process of structural reorganization. Among them, Topic_4 exhibited the highest information dispersion (71.13%), indicating that the spatial planning narrative, which dominated the early stage, experienced the greatest degree of structural adjustment and gradually lost its dominant position. In contrast, topics such as Topic_3 (25.54%), Topic_6 (23.60%), and Topic_5 (17.77%) became important driving forces in the restructuring of place identity narratives during the late stage. A single theme no longer dominates information priority; instead, it achieves a stable distribution across multiple themes. The primary function of identity narratives in this phase shifts toward narrative integration and brand consolidation. Through repeated invocation and recombination of existing thematic elements, the textual system continuously reinforces specific narrative pathways and symbolic imagery. This process compresses place identity from diverse information sources into a highly recognizable narrative framework, transforming it from “interpreted” to “recognized” and “consumed”.

## 5. Discussion

### 5.1 Theme division and information structure generation at different stages

The narrative of place identity for Harbin’s Chinese Baroque Historic Area did not originate from a single-phase communication strategy or explicit brand design. Instead, it emerged gradually through sustained selection, reorganization, and structural adjustments of thematic information elements across multiple phases of textual practice. Building upon this foundation, it is necessary to examine the functional roles different themes have assumed within the long-term textual information structure from a comprehensive perspective.

To more clearly summarize the phased division of themes within the long-term textual information structure, this study synthesizes the core semantic focus, peak prominence phases, and functional roles of each theme based on the aforementioned results analysis, as shown in Table 9.

**Table 9:** Functional roles of different topics in the long-term textual information structure.

ID	Core semantic focus	Stage of peak salience	Functional role in information structure
Topic_4	Spatial planning, cultural scenes, and symbolic places	Early	Structural anchor
Topic_1	Construction, planning milestones	Middle	Transitional amplifier
Topic_2	Historic architecture & courtyards	Middle	Narrative enrichment
Topic_3	Experience, tourism, image	Late	Branding core
Topic_5	Everyday life, tradition	Late	Identity stabilization
Topic_6	Branding, promotion, events	Late	Visibility amplifier (or Branding reinforcement)

Different themes exhibit distinct functional divisions within the long-term textual information structure. Themes centered on spatial planning, cultural scenarios, and symbolic sites primarily serve as structural anchors within the textual system, providing stable spatial boundaries and semantic frameworks for place identity narratives. Themes related to construction processes and governance implementation function as transitional amplifiers and narrative enrichers in long-term textual practices, facilitating the translation of institutional and spatial information into narrative expressions. Concurrently, themes related to experience, tourism, daily life, and brand promotion have secured stable positions within the information structure. They respectively fulfill functions such as branding, identity stabilization, and visibility amplification. Through high-frequency invocation and dissemination, these themes reinforce the overall consistency and public recognizability of place identity narratives.

However, it should be noted that the information functions identified above do not represent the entirety of place identity experiences. Rather, they primarily reflect the dominant place identity framework constructed and reinforced through institutionalized texts during the place branding process of the historic district. In other words, the information structure identified in this study reveals which place meanings were prioritized, organized, and disseminated during specific historical stages, rather than providing a complete representation of all social groups’ place identities, everyday life experiences, and spatial perceptions. Place identity is not directly produced by a single actor or a single textual system; instead, it emerges through continuous interaction and negotiation among planners, government agencies, operating entities, residents, visitors, and other stakeholders. Therefore, textual information structures should be understood as a dominant form of representation within the process of place identity construction, reflecting how particular actors assign meanings to space, reinforce cultural symbols, and shape place images through textual practices.

It is important to emphasize that the aforementioned functional roles are not mutually exclusive or linearly substitutable. Instead, they operate synergistically through dynamic adjustments in information priority and structural positioning within long-term textual practices. Most themes persist across different stages, with their evolution primarily manifesting as shifts in functional roles and structural placement rather than the emergence or disappearance of the themes themselves. For example, although the spatial planning-related theme declined in weight during the later stages, the historical spaces and cultural symbols embedded within this theme continued to serve as important foundations of place identity narratives. Meanwhile, themes related to commerce, experience, and brand communication represented further extensions and reorganizations built upon the existing spatial meanings. This functional division provides a structural foundation for understanding how place identity narratives gradually emerge and stabilize within the information architecture of long-term texts.

However, the stabilization of textual structures does not imply the disappearance of alternative interpretations of place meanings, nor does it indicate that a particular place identity narrative has acquired exclusive legitimacy. Rather, it reflects a process in which certain place meanings gain greater visibility, stronger communicative advantages, and more stable capacities for reproduction within specific institutional contexts, policy orientations, and market communication systems. Meanwhile, residents’ everyday experiences, visitors’ spatial perceptions, and commercial actors’ practices may reinterpret, adjust, or even diverge from the dominant narrative. This indicates that place identity is not unidirectionally determined by textual production; instead, it emerges through a continuous process of interaction and negotiation among narrative production, spatial practices, and social reception.

### 5.2 The progressive evolution of information priority and structural dominance: From structural foundation to narrative reinforcement

The phased division of labor among different themes within the long-term textual information structure is not accidental. It is closely tied to the informational functions that texts at different stages of the overall information system fulfill. As a continuously generated textual information system, planning texts, news texts, and propaganda texts do not replace one another. Instead, they progressively assume functions such as structural grounding, information translation, and narrative reinforcement. It should be noted that the textual information system examined in this study mainly captures the narrative production process of institutional actors involved in place branding, including governmental agencies, planning institutions, and operating organizations. These actors play critical roles in defining spatial meanings, organizing cultural representations, and communicating place images through formal textual practices. However, such institutionalized narratives represent only one dimension of place identity construction and do not fully encompass grassroots interpretations, everyday experiences, or public perceptions.

In the early stages, planning designs and policy documents systematically define the spatial boundaries, functional attributes, and cultural positioning of historic districts through institutionalized language. Their core function is to transform complex spatial and historical entities into information objects that are recognizable and manageable by institutional systems. This phase exhibits a high concentration of information priority, reflecting the textual system’s primary demand for structural stability. This process was also closely connected with concrete spatial practices and professional actors’ interpretations of place. For example, during the planning stage of the Chinese Baroque Historic Area, planners attempted to reinterpret existing cultural resources through the introduction of European-style spatial forms, plaza systems, and new functional organizations. This demonstrates that place identity narratives were not only expressed through texts but were also embedded in processes of spatial transformation and material reconstruction. Nevertheless, residents’ diverse experiences of deteriorated living conditions before renewal and improved infrastructure after renewal indicate that meanings generated through spatial transformation were not necessarily identical to planners’ intended narratives, but were continuously reinterpreted through everyday experiences.

With the intervention of news reporting and promotional texts, the informational function of textual systems gradually shifts from structural definition to information translation. Existing institutional and spatial information is reorganized into more narrative and contextual forms of expression, enabling its entry into public communication contexts. Correspondingly, information priorities are redistributed across multiple themes, and textual structures transition from single-dominant narratives to the coexistence of multiple themes. During this stage, commercial operators and spatial users gradually participated in the production of place meanings. For example, merchants’ descriptions of traditional clothing experiences, nostalgic street atmospheres, and film-related scenes demonstrate the transformation of the historic district from a planned cultural space into an experiential consumption space. However, these everyday practices did not always completely follow official branding narratives, but were continuously adjusted and negotiated among commercial demands, tourist experiences, and local memories. Therefore, the formation of place identity includes not only top-down narrative production but also the reinterpretation and negotiation of spatial meanings by different actors.

In the later stages, promotional texts and new media texts build upon existing information structures while further emphasizing experiential narratives, visual imagery, and the repeated presentation of symbolic meanings. The function of these texts shifts from explanatory to dissemination-oriented, with certain high-salience themes securing more stable positions within the structure. This drives place identity narratives toward convergence within recognizable, communicable narrative frameworks. The increasing importance of tourism experiences, cultural activities, and visual consumption reflects the transformation of place identity from institutional definition toward publicly recognizable images. However, these processes do not imply passive acceptance of official narratives. Instead, visitor participation, online communication, and everyday spatial practices contribute to the continuous reproduction, adjustment, and reinterpretation of place meanings.

Therefore, it is evident that the shift from information priority to structural dominance does not result from a linear replacement but rather reflects the progressive layering of information functions across different stages. This progressive process provides a functional-level explanation of the convergence trend in information structures within long-term textual systems.

### 5.3 Relational patterns and the information-structural outcome

To further validate whether the aforementioned functional evolution manifests as stable information structural configurations at the relational level, this study introduces a keyword co-occurrence network analysis. This builds upon the completed thematic identification, information priority, and structural dominance analyses to supplement the examination of relational patterns among different thematic information elements within long-term textual systems. This analysis does not aim to identify new thematic types but rather to investigate whether different information elements form holistic structural configurations through sustained co-occurrence. This explores whether information structures exhibit holistic organizational characteristics at the relational level—beyond simple thematic juxtaposition—and provides relational support for understanding place identity narratives as information-structural outcomes.

Fig 7 presents a co-occurrence network constructed based on high-frequency keywords related to thematic elements. This network examines the overall structural form of information components in the identity-narrative texts of the Chinese Baroque Historic Area from a relational perspective. Overall, the network exhibits highly connected, densely interrelated characteristics, with multiple connections between different information components. No isolated or clearly segmented subgroups have formed. This finding indicates that thematic information within the Chinese Baroque identity narrative does not exist as mutually independent semantic units. Instead, through repeated co-occurrence and combination during prolonged, multi-stage textual practices, it progressively forms a highly coupled information structure. This relational pattern structurally supports this study’s analytical perspective, which interprets place identity narratives as structural outcomes of information rather than simple thematic overlays.

**Fig 7:**
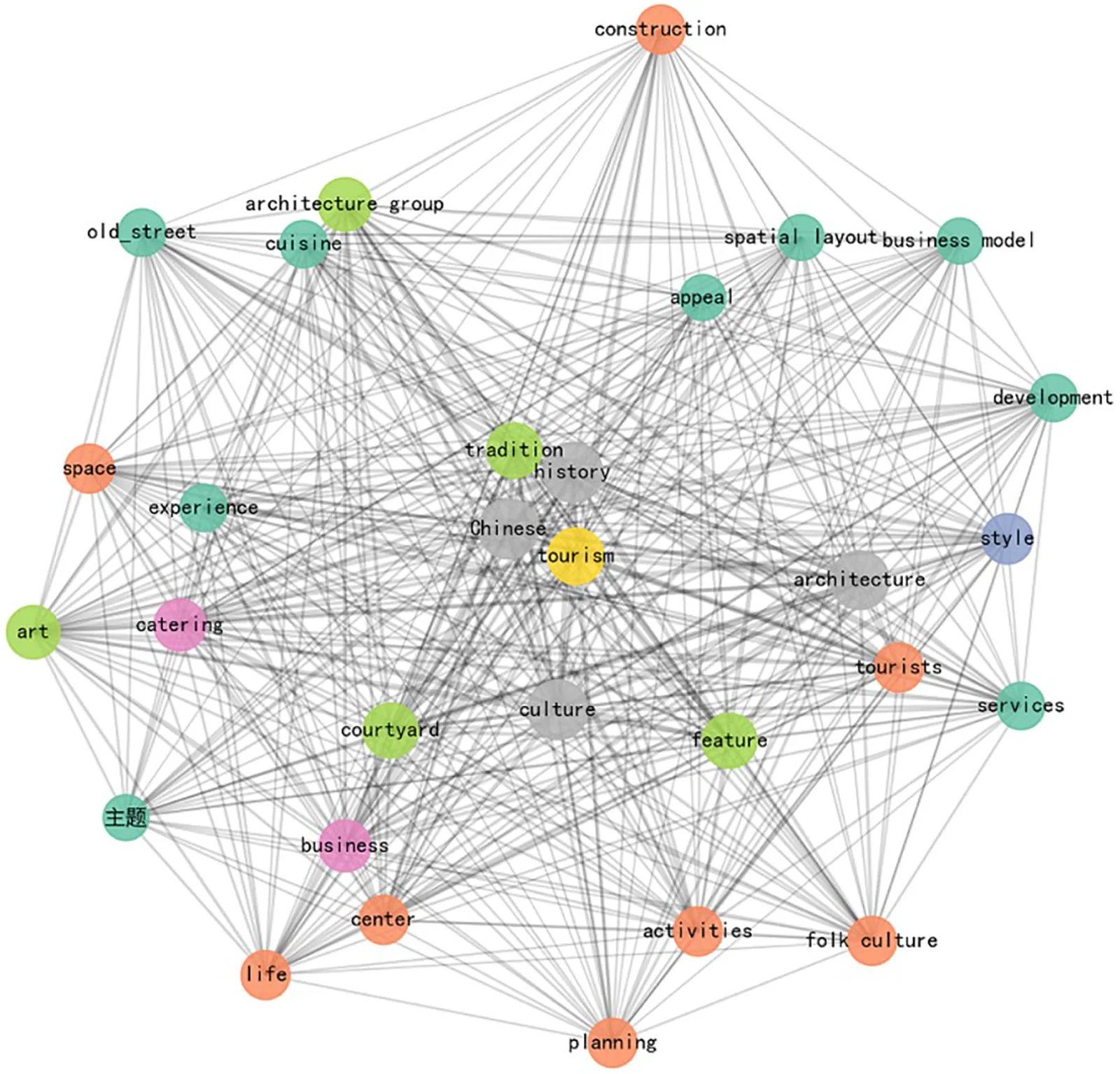
Keyword co-occurrence network of place identity narratives in the long-term textual system.

Based on the network’s spatial distribution, keywords related to history, culture, and tradition occupy relatively central positions, forming high-frequency connections with diverse information elements such as planning, space, commerce, experience, and art. These information elements are consistently invoked across different themes and textual phases, demonstrating pronounced cross-thematic integration and serving as structural hubs within the overall information architecture. They do not merely serve a single narrative dimension but establish stable semantic connections between institutional definitions, spatial expressions, and experiential narratives, providing a continuously accessible core information foundation for place identity narratives. However, the central position of keywords within the textual network does not indicate that textual narratives alone determine place identity. Instead, it reflects the symbolic resources selectively emphasized and strengthened during the branding process. These symbolic resources are not fixed meanings but are continuously negotiated and reinterpreted through spatial practices, commercial activities, residents’ experiences, and tourists’ perceptions.

In contrast, information elements related to planning implementation, construction management, commercial operations, event organization, and service provision are more widely distributed across the network’s peripheral areas. They are primarily embedded within the overall structure through connections to the aforementioned core cultural-historical elements. This indicates that such functional and operational information does not, in itself, constitute the core content of identity narratives. Instead, it relies on existing cultural and historical imagery to be endowed with phased narrative functions and communication objectives. Its position within the network reflects the functional roles—such as information translation, narrative expansion, and communication reinforcement—assumed by planning texts, news texts, and promotional texts at different stages.

It is important to emphasize that this keyword co-occurrence network does not exhibit clear structural segmentation based on thematic units. Instead, it manifests as the overlapping coexistence of multiple categories of informational elements within the same relational space. This relational structure indicates that the information evolution of the Chinese Baroque identity narrative is not achieved through linear thematic replacement or phased “creation”. Rather, it gradually forms relatively stable structural configurations through the continuous adjustment and reorganization of relational intensities among informational elements across textual practices spanning different phases. As the function of texts gradually shifted from institutional definition to public communication and image-building, the information structure evolved over the long term from a state of high uncertainty to a relatively stable configuration.

Based on the aforementioned thematic role division and progressive information functionality, this study further proposes a “stage-based information compression mechanism” to characterize the intrinsic process by which information structures evolve from instability toward relative stability within long-term textual information systems. It is crucial to emphasize that the information compression referred to herein does not imply simplification of semantic content or reduction in overall information volume. Rather, it denotes the structural convergence trend exhibited in the distribution of information priority across comparative stages. As textual practices advance, the highly concentrated structural dominance observed in early stages gradually diminishes. Differences in information priority among themes continuously narrow, and the overall fluctuation range of thematic weight substantially decreases. This mechanism indicates that within the specific case of the Chinese Baroque Historic Area, the stabilization of place identity narratives is not achieved through linear replacement of themes or phased “creation”. Most core themes persist across different stages, with their evolution primarily manifesting as dynamic adjustments in relative priority and structural roles. Through the progressive layering of informational functions across different stages, the long-term textual practices examined in this study contributed to the gradual integration and stabilization of the place identity information structure. Therefore, rather than suggesting a universal evolutionary pathway applicable to all place branding processes, this case demonstrates how information structures may become gradually organized and stabilized under specific historical, institutional, and spatial conditions.

## 6. Conclusions

This study analyzes the Chinese Baroque Historic Area in Harbin, China, systematically examining the information structure characteristics and evolutionary mechanisms of place identity narratives within long-term texts. This analysis is based on planning documents, news reports, and promotional materials formed across different stages. The main conclusions are as follows:

(1) The textual materials concerning the Chinese Baroque Historic Area have evolved over a long, multi-stage process, forming a distinct phased information structure. This structure is neither static nor uniformly distributed; rather, it exhibits a dynamic progression from high concentration to the coexistence of multiple themes, and ultimately toward relative equilibrium. In the early stage, planning and policy documents were dominated by a single institutional theme. During the middle stage, information priorities were redistributed among multiple themes. In the late stage, experience- and image-oriented themes became the structural core, leading to a stabilization of the information structure.
(2) Narrative texts at different stages serve distinct and progressively interconnected informational functions within place identity narratives. Early texts primarily establish informational structures and define semantic frameworks. Middle stage texts perform information translation, converting institutional information into narrative expressions suitable for public dissemination. Late texts drive the convergence of place identity toward stable, communicable narrative frameworks through narrative integration and reinforcement.
(3) The reorganization of thematic information elements across stages exhibits non-linear evolutionary characteristics. Most core themes persist across different phases, with their evolution primarily manifested through dynamic adjustments in information priority and structural dominance rather than linear replacement. As the stages progress, the dominance of any single theme gradually diminishes, and the weight differences between themes converge, reflecting non-linear structural stability achieved through a phased information-compression mechanism.

Overall, this study conceptualizes the identity narrative texts of Harbin’s Chinese Baroque Historic Area as a long-term textual information system and examines how place identity narratives evolve during historic district branding processes. By integrating the Latent Dirichlet Allocation (LDA) topic model with qualitative research methods, the study systematically examined the informational characteristics of place branding narrative texts across dimensions of information priority, structural dominance, and dispersion. The findings demonstrate that, within the specific historical and institutional context of this case, narratives of place identity are not fixed representations of place but dynamic information structures continuously shaped through textual production, spatial practices, and communication processes. Place identity narratives continuously evolve through adjustments in information priority and restructuring of structural relationships, with the information structure gradually forming relatively stable patterns over time. Such stabilization does not imply the fixation of place meanings, but rather reflects the process through which certain meanings gain greater visibility, institutional support, and communicative advantages within specific branding contexts. Rather than providing universally generalizable conclusions or direct prescriptions for urban renewal practices, these findings offer an analytical framework and empirical reference for understanding how information structures of place identity narratives are selected, reorganized, and stabilized within comparable historic district branding contexts.

It should be noted that the LDA thematic model employed in this study primarily reveals the generation and evolution of narrative identities within place branding texts. However, its analysis remains focused on the textual structural level, with relatively limited capacity for deep interpretation of local historical and cultural connotations, as well as for explaining how different stakeholders understand and perceive place identity narratives. The present study primarily elucidates the mechanisms through which dominant narratives of place identity are formed during the place-branding process, but does not systematically incorporate the diverse perspectives and expressions of grassroots actors. Future research could further integrate institutional texts, grassroots narratives, and spatial behavioral data to examine how place identity narratives are negotiated, contested, and reconstructed across the dimensions of production, circulation, and reception. Given that this study focuses on a single historic district, future research should conduct comparative studies across different spatial scales, cultural contexts, and institutional environments to examine whether similar information-structural patterns exist under different place branding conditions and to further evaluate the transferability of the proposed analytical framework. Concurrently, incorporating network analysis and other machine learning methods could provide cross-validation for the robustness of internal information structure variations within place identity narratives. Furthermore, structural differences at the textual level do not necessarily correlate with disparities in brand communication effectiveness. Subsequent research should integrate communication data, urban development indicators, and qualitative interview materials to deepen understanding of the mechanisms governing place branding formation and dissemination across diverse institutional contexts, thereby providing theoretical underpinnings for advancing city marketing and sales strategies.

## Supporting information

S1 Table Metadata of textual documents used for corpus construction. (PDF)

S2 Table LDA topic modeling outputs. (PDF)

S3 Table Information structure indicators across different stages. (PDF)
