# Supplementary material for: The evolution of place identity narratives: Long-term textual practices in the Chinese Baroque Historic Area
Source: PLoS One. 2026 Oct 5;21(10):e0359404. doi: 10.1371/journal.pone.0359404 (PMC13637921; doi:10.1371/journal.pone.0359404)
Supplement: Supplementary file 1 [file pone.0359404.s001.pdf]

S1 Table. Metadata of textual documents used for corpus construction.

Text No.	Title	Year	Stage	Source
T01	The Original Appearance of the Chinese Baroque Historic Area	2008	Early Stage (Planning and Construction Stage)	News Media
T02	Completion of the Planning Scheme for the Daowai “Chinese Baroque” Renovation Demonstration District	2007		News Media
T03	Project Description of the Renovation and Regeneration of the Chinese Baroque Historic Area	2012		Harbin Municipal Planning Authority
T04	Harbin’s “Chinese Baroque” Buildings Undergo Conservation and Regeneration	2008		News Media
T05	Restoration and Regeneration of Historic Districts: A Study on Historic Buildings	2008		Harbin Municipal Planning Authority
T06	Narration Script for the Chinese Baroque Historic and Cultural Area	2013	Middle Stage (Activation and Utilization Stage)	News Media
T07	The Chinese Baroque Historic Area Scheduled to Officially Open in May, Becoming a New Landmark of Harbin	2012		News Media
T08	Planning Consultant Report for the Harbin Chinese Baroque Historic Area	2012		District Operation Company
T09	The Main Construction of the Second-Phase Renovation Project of Harbin Chinese Baroque Historic Area Completed in October	2010		News Media
T10	Investment Promotion Brochure for the Chinese Baroque Cultural Tourism Commercial District	2012		District Operation Company
T11	Tourism Guide to Laodaowai Chinese Baroque Historic Area	2015		District Operation Company
T12	Reconfiguration of the Chinese Baroque Historic and Cultural Conservation District	2010		News Media
T13	Introduction to Laodaowai Chinese Baroque Historic Area	2015		News Media
T14	Phase II of the Chinese Baroque Historic Area Completed in October: Four Zones Reconstruct the Everyday Urban Culture of Old Daowai	2012		News Media
T15	The Chinese Baroque Historic Area Project Is Expected to Open to the Public in October	2013		News Media
T16	Exploring the Chinese Baroque Historic and Cultural Area: Cultural Renewal and Growing Visitor Vitality	2024	Late Stage (Marketing and Promotion Stage)	News Media
T17	Recommendations for Branding and Promotion Strategies of Chinese Baroque	2021		District Operation Company
T18	Hand-drawn Tourist Map of the Chinese Baroque Historic Area	2024		District Operation Company
T19	Marketing Brochure of the Chinese Baroque Historic Area	2024		District Operation Company
T20	Promotional leaflet for the Chinese Baroque Historic Area	2024		District Operation Company
T21	Promotional Brochure of the Chinese Baroque Historic Area	2021		District Operation Company
T22	A Slow-Living Experience at the Chinese Baroque Historic Area	2023		News Media
T23	Chinese Baroque Historic Area: A Showcase of Chinese-Western Integrated Architectural Art	2024		News Media
T24	Outcomes of Investment Attraction in the Chinese Baroque Historic Area	2024		District Operation Company

Note: “Year” indicates the publication or production year, whereas “Stage” reflects the document’s primary practical function and substantive content rather than publication year alone.
