# Supplementary material for: The evolution of place identity narratives: Long-term textual practices in the Chinese Baroque Historic Area
Source: PLoS One. 2026 Oct 5;21(10):e0359404. doi: 10.1371/journal.pone.0359404 (PMC13637921; doi:10.1371/journal.pone.0359404)
Supplement: Supplementary file 2 [file pone.0359404.s002.pdf]

S2 Table. LDA topic modeling outputs.

Text No.	Stage	Topic_0 (%)	Topic_1 (%)	Topic_2 (%)	Topic_3 (%)	Topic_4 (%)	Topic_5 (%)	Topic_6 (%)
T01	Early Stage (Planning and Construction Stage)	0.16	0.16	0.16	0.16	99.04	0.16	0.16
T02		0.13	0.13	0.13	0.13	99.24	0.13	0.13
T03		0.03	0.03	0.03	0.03	99.82	0.03	0.03
T04		0.15	0.15	0.15	99.09	0.15	0.15	0.15
T05		0.09	0.09	0.09	0.09	99.49	0.09	0.09
T06	Middle Stage (Activation and Utilization Stage)	0.01	0.01	99.96	0.01	0.01	0.01	0.01
T07		0.10	99.39	0.10	0.10	0.10	0.10	0.10
T08		99.99	0.00	0.00	0.00	0.00	0.00	0.00
T09		0.31	10.37	0.31	0.31	77.78	0.31	10.62
T10		0.01	99.93	0.01	0.01	0.01	0.01	0.01
T11		0.02	0.02	0.02	0.02	34.94	64.93	0.02
T12		0.05	30.39	0.05	0.05	69.37	0.05	0.05
T13		0.31	10.37	0.31	0.31	77.78	0.31	10.62
T14		0.12	16.38	0.12	0.12	83.02	0.12	0.12
T15		0.09	0.09	0.09	0.09	99.48	0.09	0.09
T16	Late Stage (Marketing and Promotion Stage)	0.05	0.05	0.05	99.71	0.05	0.05	0.05
T17		0.02	0.02	40.32	0.02	37.60	22.00	0.02
T18		0.03	0.03	68.85	0.03	0.03	29.45	1.60
T19		0.03	92.39	0.03	0.03	0.03	0.03	7.47
T20		0.03	0.03	0.03	0.03	32.68	67.18	0.03
T21		0.02	0.02	0.02	0.02	0.02	0.02	99.89
T22		0.02	17.09	0.02	30.9	5.29	42.15	4.52
T23		0.05	0.05	0.05	99.73	0.05	0.05	0.05
T24		0.04	0.04	0.04	0.04	0.04	0.04	99.77
