# Supplementary material for: The evolution of place identity narratives: Long-term textual practices in the Chinese Baroque Historic Area
Source: PLoS One. 2026 Oct 5;21(10):e0359404. doi: 10.1371/journal.pone.0359404 (PMC13637921; doi:10.1371/journal.pone.0359404)
Supplement: Supplementary file 3 [file pone.0359404.s003.pdf]

S3 Table. Information structure indicators across different stages.

Stage	Information priority	Information priority (%)	Structural dominance	Shannon entropy	Information compression
Early Stage (Planning and Construction Stage)	Topic_4	79.55	0.67	0.54	0.72
Middle Stage (Activation and Utilization Stage)	Topic_4	41.70	0.29	1.42	0.27
Late Stage (Marketing and Promotion Stage)	Topic_3	25.61	0.19	1.72	0.12
